# Effect of VH–VL Families in Pertuzumab and Trastuzumab Recombinant Production, Her2 and FcγIIA Binding

**DOI:** 10.3389/fimmu.2018.00469

**Published:** 2018-03-12

**Authors:** Wei-Li Ling, Wai-Heng Lua, Jun-Jie Poh, Joshua Yi Yeo, David Philip Lane, Samuel Ken-En Gan

**Affiliations:** ^1^Bioinformatics Institute, Agency for Science, Technology and Research (A*STAR), Singapore, Singapore; ^2^p53 Laboratory, Agency for Science, Technology and Research (A*STAR), Singapore, Singapore

**Keywords:** complementary determining region grafting, FcγIIA, *K*_D_, Pertuzumab, recombinant antibody production, Trastuzumab, therapeutic antibodies, VH–VL families

## Abstract

Many therapeutic antibodies are humanized from animal sources. In the humanization process, complementarity determining region grafting is tedious and highly prone to failure. With seven known VH families, and up to six known κ VL families, there are choices aplenty. However, the functions of these families remain largely enigmatic. To study the role of these V-region families, we made 84 recombinant combinations of the various VH and VL family whole IgG1 variants of both Trastuzumab and Pertuzumab. We managed to purify 66 of these to investigate the biophysical characteristics: recombinant protein production, and both Her2 and FcγIIA binding. Our findings revealed combinations that showed improved recombinant antibody production and both antigen and receptor binding kinetics. These findings show the need to rethink antibodies as a whole protein, relooking of the functions of the antibody domains, and the need to include immunoglobulin receptor investigations for effective antibody therapeutics development.

## Introduction

Human antibodies or immunoglobulins (Igs) are typically made up of two symmetrical arms comprising of a heavy (H, that includes μ or M, α or A, δ or D, γ or G, ε or E constant regions) and a light (L, that includes the λ or κ families) chains encoded in human chromosomes 14, 22, and 2, respectively (1, 2). The heavy chain locus contains diverse genes of variable (V), diversity (D), joining (J), and constant (C) regions/domains while the light chain locus contain diverse genes of variable (V), joining (J), and constant (C) regions/domains. These loci undergo V(D)J recombination to produce variations of three antigen interacting loops known as complementarity determining regions (CDRs) that are supported by scaffolds known as framework regions (FWRs). These CDRs, widely recognized for binding and recognition of the vast spectrum of antigens, are the focus for grafting during antibody humanization. Current antibody investigations typically focus on the VH FWRs, neglecting the VL FWRs. Within the VH families, certain FWR families are associated with allergy or autoimmune diseases. For example, VH1 is found overrepresented in peanut allergies (3), VH5, in allergic rhinitis (4), and VH3 and VH4 are found overrepresented in certain microbial infections (5–7). In addition, many of these VH FWRs are also involved in interacting with superantigens such as proteins A and G (8, 9) whereas only specific Vκ FWR families (10) are known to interact with protein L.

Typically, therapeutic monoclonal antibodies (mAbs) would flag antigens for immune cells engagement. At the point of writing, most therapeutic anti-bodies are of the IgG isotype (11), engaging the immune effector cells via the FcγR on the surface of these cells. Among the known Fcγ receptors (R), FcγRIIA, also known as CD32, has the ability to bind to all the four human subtypes of IgG, and is the most prevalent, being found on most immune cells types (macrophages, neutrophils, eosinophils, platelets, and Langerhans cells) (12). FcγRIIA is also unique from the rest of the FcγR in that it binds IgG antibodies at the lower hinge and adjacent region of CH2, closer to the V-region (13), possibly making it more sensitive to FWR and CDR changes. In this aspect, the influence of V-regions (specifically the FWRs) of whole Igs on FcR (e.g., FcγRIIA) engagement remains uncharacterized.

In our previous work, we showed that deletions alone in the FWRs region affected recombinant production and antigen-binding affinity (14). Together with our antibody class swap experiments (15), we further demonstrated that the heavy chain constant regions can have effects on antigen binding. These shows that the various antibody regions would have wide-ranging effects beyond localized functions and raises the possibility that the reverse, where V-region changes may have effects on the constant regions, particularly on the FcR binding may occur. While there are existing studies showing the pairing effects of various V-region FWR families, including that to the different germlines within the families [e.g., Ref. (16)], such investigations often do not optimize whole antibodies, thereby providing limited provision to investigating whole therapeutic antibodies. This is especially so in the very crucial areas of antigen binding and FcR engagement.

**Figure 1:**
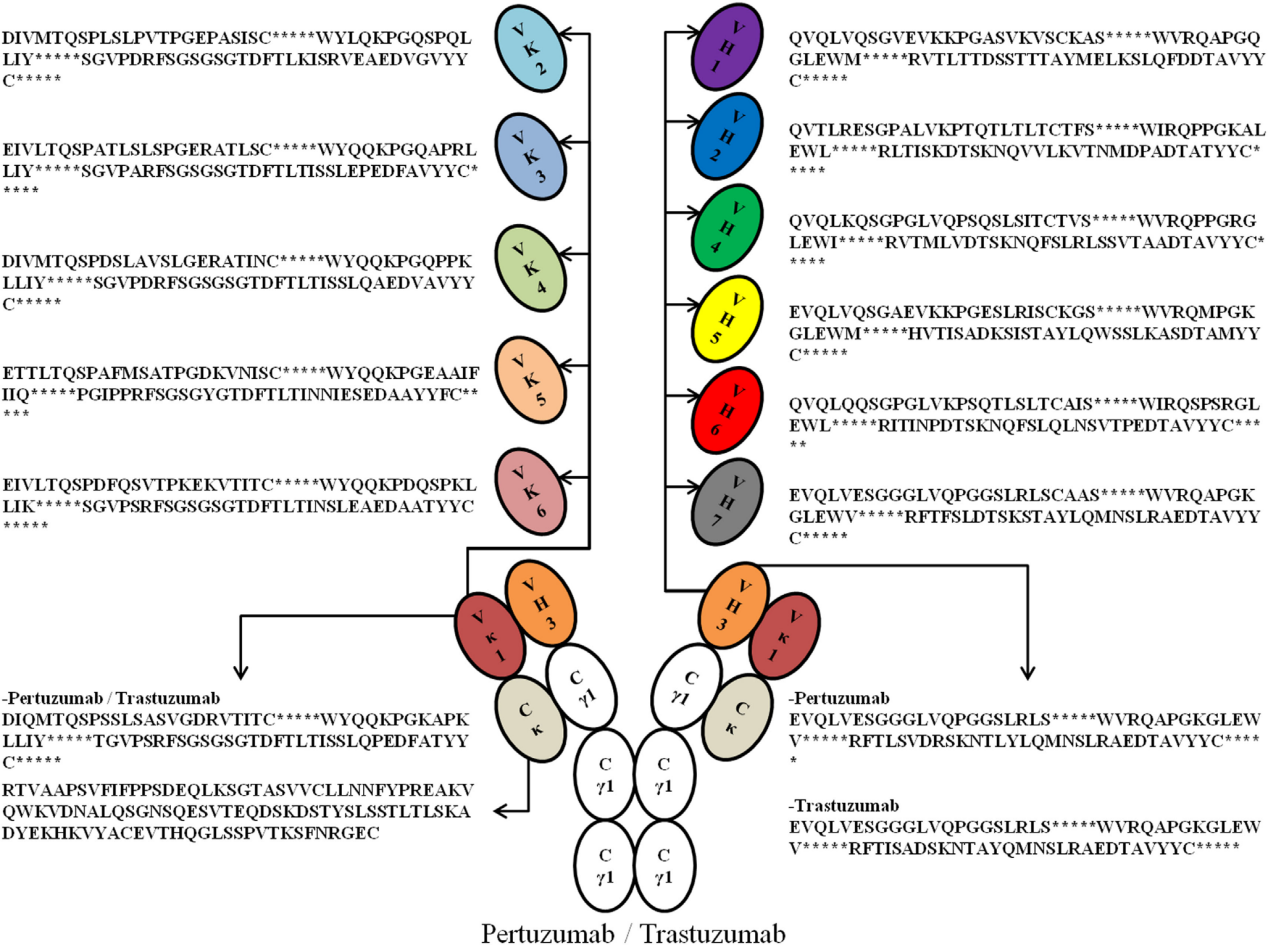
An illustration of the possible permutations of all the Vκ (1–6) and VH (1–7) families with framework regions sequences used in this study. *Denotes the complementarity determining regions (CDRs). For fully grafted sequences with CDRs, refer to Data S1 in Supplementary Material.

To study the crucial effects of antibody VH–VL FWRs on antigen binding and receptor engagement, we grafted the respective VH and VL CDRs of previously humanized murine antibodies: Trastuzumab and Pertuzumab, into six Vκ (Vκ1–6) FWRs (we excluded Vλ FWRs to reduce confounding factors due to differences in physicochemical properties as reported in Ref. (17) and that both antibodies are originally Vκ) and seven VH FWRs (VH1–7) identified by the international ImMunoGeneTics information system (IMGT) (18), thereby forming 42 possible pairing combinations. Using a double plasmid transient transfection method, we obtained an extensive permutation of 84 whole IgG1 variants of Trastuzumab and Pertuzumab (Figure 1 for illustration and sequences).

The antibodies were then characterized with respect to recombinant antibody production, and their binding kinetics (association and dissociation rates) to the known Her2 antigen and the human IgG receptor FcγRIIA were measured. Through these variants, we were able to 1) establish a template list of VH and Vκ FWRs that could be used for humanizing and modifications of therapeutic antibodies; 2) establish the effects of these FWRs on antigen and receptor engagement; and 3) optimize the best Trastuzumab and Pertuzumab VH|VL pairing for both antigen and receptor binding.

## Materials and Methods

### Trastuzumab and Pertuzumab CDR Grafting

**Figure 2:**
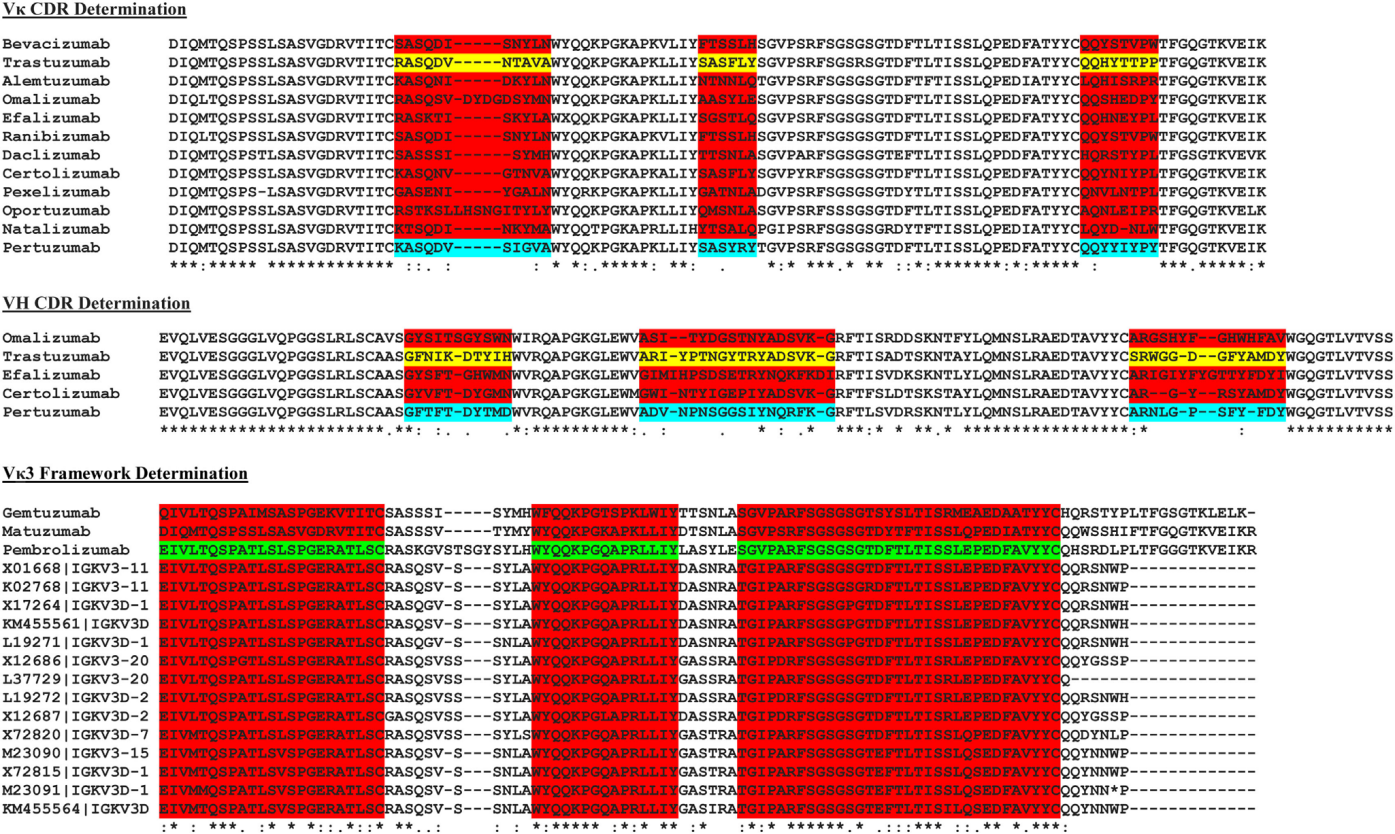
The multiple sequence alignment of known commercial antibodies s used for determining the light (top) and heavy chain (middle) complementarity determining regions (CDRs). An example showing Vκ3 framework region determination is as shown (bottom). For the full list, refer to Data S2–S16 in Supplementary Material.

The sequences of a number of known commercial mAbs (Figure 2) were subjected to IMGT V-Quest (19) analysis to determine the VH and VL families followed by alignment to the Trastuzumab (PDB 1N8Z) and Pertuzumab (PDB 1S78) sequences to determine the CDRs of both antibody heavy and light chains. The amino acid sequences of these commercial mAbs were also sorted according to their respective VH and VL families and compared with the corresponding VH and VL sequence database in IMGT (18) to establish the FWRs.

Final FWRs sequences (Figure 1) for grafting were chosen based on prevalence in its respective family (Data S4–S16 in Supplementary Material).

The following frameworks were derived from commercial antibody sequences: Vκ1, Vκ3, VH1, VH2, VH3, VH4, and VH7; whereas the rest were grafted into the most prevalent germline sequences in the respective families from the IMGT database.

### Production of the Various Antibody VL and VH Chains and FcγIIA

Vκ and VH genes were synthesized (by Integrated DNA Technologies) with silent restriction enzyme sites and an immunoglobulin (Ig) leader incorporated as previously described (20). Briefly, an EcoRI site was added before the signal peptide and an ApaI as a silent mutation was introduced to the VH-C region. For the VL families, an EcoRI was added to the 5′ site of the Ig leader and a KpnI site at the J-chain. The VH genes were ligated into the pTT5 vector (Youbio) already holding the human IgG1 constant (15) while the Vκ genes were ligated into the pTT5 vector with the κ-constant region. The extracellular portion of the human FcγIIA (Accession no. NP_001129691, 1aa-215aa) was also synthesized, but with the addition of 3′ 10× HIS tag. It was ligated into the pTT5 plasmid using EcoRI and HindIII restriction sites.

The completed plasmids were prepared (21) and transformed into DH5α competent cells for plasmid production as previously described (22). The plasmids were then transfected into EXPI 293F (Invitrogen) as previously performed (14, 15, 23) with the exception of the use of low Ig fetal bovine serum (FBS, Gibco) for Ig production while normal FBS (Gibco) was used for FcγIIA production. For transfection ratio tests, varying DNA ratios (Vκ:VH) of 1:1, 1:3, and 3:1 where total amount of DNA was maintained at 1 µg/ml in 6-well plates were used as previously described (20). Larger scale antibody productions for purifications were performed at 1:1 L:H ratio, scaling up the cell number and transfection agent proportionally. FcγIIA single plasmids were also transfected at 1 µg/ml concentrations in the same proportions.

### Recombinant Protein Purification

The supernatant of the transfected cell cultures were collected a fortnight later and spun down at 4,000 rpm, 4°C, for 1 h before 0.2 µm filtration and purified using the ÄKTA pure system (GE Healthcare) using Protein G affinity column for the IgGs as previously described (15). The 5 ml HiTrap TALON crude affinity column (GE Healthcare) was used for the recombinant FcγIIA using the same settings. The affinity purified proteins were subsequently gel filtrated using the Superdex 200 pg 16/60 column (GE Healthcare) precalibrated with Gel filtration HMW calibration kit (GE Healthcare) and the Gel filtration LMW calibration kit (GE Healthcare) to extract the monomeric fractions as previously described (14, 15). Pure IgG1 variants and FcγIIA fractions were collected and concentrated using 100 kDa Amicon Pro System (Merck) and 10 kDa Amicon Pro System (Merck) concentrators, respectively. The final concentrations of the proteins were determined by spectrophotometric analyses using nanodrop (Thermo Fisher Scientific) with consideration of calculated protein extinction coefficients of the various IgG1 variants and FcγIIA.

### Recombinant Antibody Quantification

The Octet QK^e^ system (ForteBio) was used to quantify the amount of antibodies in transiently transfected cell cultures supernatants using Protein G biosensors (ForteBio) with preloaded program settings (high sensitivity assay with regeneration) in Octet Data Acquisition 7.0 as previously described (14, 15).

### Binding Measurements

The rate of association (*K*_on_) and rate of dissociation (*K*_off_) of the antibody variants to Her2 were measured using Anti-Human Fc Capture (AHC, ForteBio) to capture the antibody followed by measurement of Her2 binding using the Octet QK^e^ system with the Octet Data Acquisition 7.0 software as performed previously (14, 15). Briefly, the programs are as followed: preconditioning; initial baseline; loading of antibodies; baseline; association to Her2; dissociation from Her2; and stripping. The duration of each step is as per manufacturer’s recommendations.

For FcγIIA binding measurements, the same software settings were performed for Her2 binding measurements with minor modification. Recharging of the Ni-NTA biosensor was performed using 10 nM of nickel chloride for 60 secs prior as per manufacturer’s recommendations between preconditioning and initial baseline. Purified FcγIIA was first bound to Ni-NTA biosensor (ForteBio) for the loading step before measurement of antibody bindings (100 to 6.25 nM) in association and dissociation steps.

The equilibrium dissociation constant (*K*_D_) was calculated from the *K*_on_ and *K*_off_ automatically by the Octet Data Acquisition 7.0 software.

## Results

### CDRs of Pertuzumab and Trastuzumab

To graft the CDRs of both Trastuzumab and Pertuzumab into the various human VH and VL FWRs, we first determined the CDRs of the original Vκ1|VH3 pairing of both antibodies by comparing their amino acid sequences with the Vκ1|VH3 families of other commercial antibodies using multiple sequence alignment. The contrasting regions were identified as CDRs while the consensus regions were defined as FWRs as shown in Figure 2.

### Determining the VL and VH FWRs for Antibody Grafting

As the exact boundaries for CDR and FWRs remain enigmatic with numerous algorithms such as Kabat, Clothia, Martin, and AHo (24–27) establishing different FWR–CDR boundaries, we performed our own multiple sequence alignment with all known VH and VL sequences found in the IMGT database (18). The most conserved regions were determined as FWRs (Figure 2) with guidance from the abovementioned algorithms.

Upon joining the FWRs and CDRs sequences, the final grafted sequences were checked using IMGT V-QUEST (19, 28) to reaffirm the family type. All input results were correctly identified by IMGT except for Pertuzumab VH6 and both Pertuzumab and Trastuzumab VH7. Since the same methodology for FWRs determination was employed for all the families, experiments proceeded despite the issues with VH6 and VH7 which were assumed to be due to biases in the IMGT algorithm.

### Effects of VL and VH FWR Families in Small Scale Recombinant Antibody Production

To investigate the effects of the VL and VH framework combinations on whole recombinant antibody production, we transfected EXPI 293 F cells with different ratios of light and heavy chain plasmids based on the 84 possible antibody combinations (42 Pertuzumab and 42 Trastuzumab combinations), normalizing the production rates with the original Pertuzumab and Trastuzumab FWR pairings (Vκ1 and VH3).

**Figure 3:**
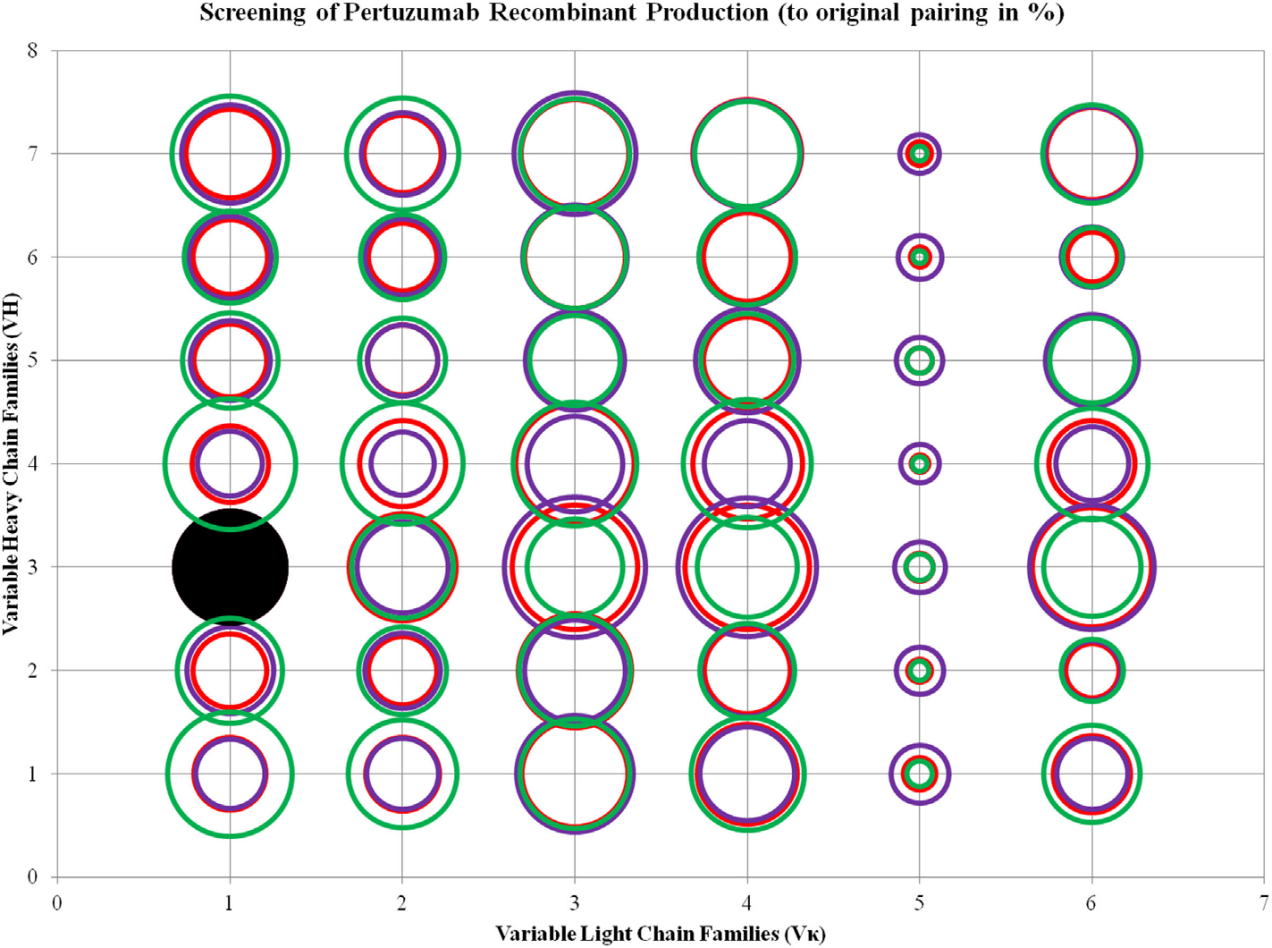
Bubble chart representations of the production of recombinant Pertuzumab antibody variants. The production levels are directly proportional to the area of the circle. The columns show the Vκ family while the rows show the VH families. Each circle in the respective combination shows one of the three plasmid ratio transfections (red, 1:1; purple, 1:3; and green, 3:1) while black solid circles show the original pairings. All circles are benchmarked against the black solid circle which is at 100%. A table of the production rates is also shown in Figure S1 in Supplementary Material.

We found trends where some VL and VH families tend to be limiting factors to overall whole IgG1 antibody production. Specific for the Pertuzumab variants, 1:1 (L:H) ratio has shown that the Vκ3|VH3 pair is the best producing pair (125.29 ± 15.91%) while the Vκ5|VH4 pair is the worst producing combination (2.47 ± 2.12%). Generally, the trends for the L chain production levels are as follows: Vκ3 > 4 > 6 > 1 > 2 > 5 while H chain production levels are as follows: VH3 > 4 > 7 > 1 > 5 > 2 > 6, regardless of the partner chains paired with. ANOVA tests between the three ratios showed that Vκ1 and 2 families as well as VH1 and 4 families were significantly different, with a trend for increased L chain plasmids to increase production levels. This was observed even when decreasing H chain amounts to keep to the same total DNA within the co-transfection experiments. While there was no specific pattern observed for H chain families acting as limiting factors, VH6 productions stood out to be relatively consistent in production (regardless of the ratio used) and were not significantly affected by the amount of L chains (Figure 3; for more details, refer to Figure S1 in Supplementary Material).

To determine if the production rates were influenced by CDRs or simply contained to the FWRs, we performed the same grafting of Trastuzumab CDRs to these VH–VL frameworks. Trastuzumab has the same VH3 and Vκ1 pairing with fairly similar CDRs as Pertuzumab and binds to the same Her2 anti-gen on a different epitope. Within the Trastuzumab combinations, Vκ4|VH3 was the best producing pair (276.50 ± 63.13%) while Vκ5|VH2 is the lowest produced pair (2.67 ± 5.14%). The trend in production levels for the VL families are as follows: Vκ4 > 3 > 6 > 2 > 1 > 5, while that for the VH families are as follows: VH 3 > 5 > 7 > 4 > 1 > 6 > 2, regardless of the paired partners.

**Figure 4:**
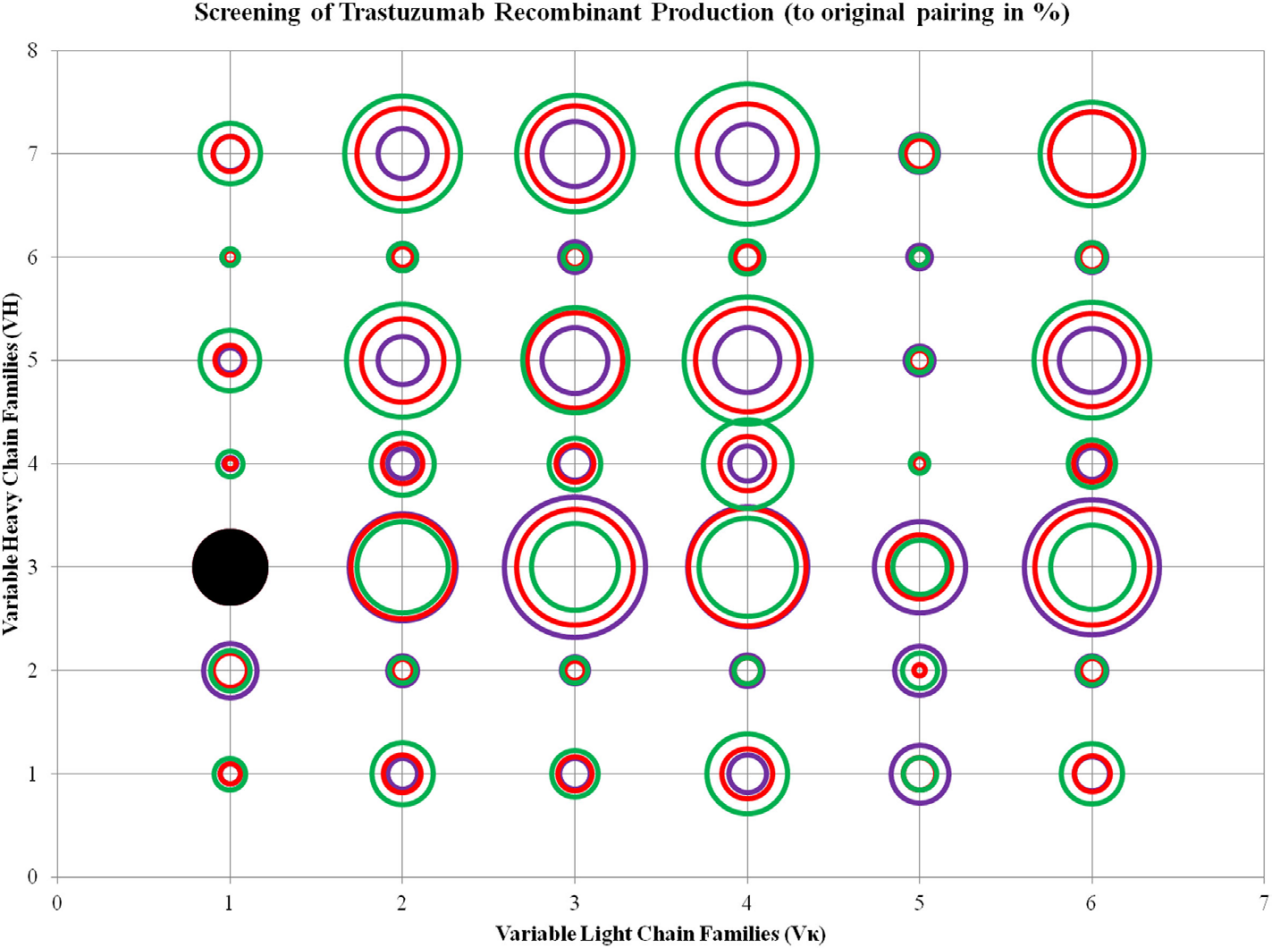
Bubble chart representations of the production of recombinant Trastuzumab antibody variants. The production levels are directly proportional to the area of the circle. The columns show the Vκ family while the rows show the VH families. Each circle in the respective combination shows one of the three plasmid ratio transfections (red, 1:1; purple, 1:3; and green, 3:1) while black solid circles show the original pairing. All circles are benchmarked against the black solid circle which is at 100%. A table of the production rates is also shown in Figure S2 in Supplementary Material.

ANOVA test performed on the three ratios of Trastuzumab transfection showed that Vκ2, 4, and 6 families, as well as VH1, 5, and 7 were significantly different, and the increase of L chain plasmids boosted production, even with decreasing H chains amounts in the same total DNA concentration. The same observation for VH6 as seen in Pertuzumab applies for Trastuzumab with the addition of VH2 also being largely independent of VL influence in production rates (Figure 4; for more details, refer to Figure S2 in Supplementary Material).

### Effects of VL and VH FWR Families on Antigen-Binding Kinetics to Her2

To determine the effects of these VL and VH framework families on antigen binding, we scaled up the production of the whole IgG1 antibodies and managed to purify 66 out of 84 combinations (Figures S3 and S4 in Supplementary Material) for size exclusion chromatography after Protein G affinity purifications to obtain pure monomeric fractions. The monomeric fractions were determined based on the elution volume (at ~70 ml) as was previously calibrated (14, 15).

To measure the association (*K*_on_) and dissociation (*K*_off_) rates of the antibodies to Her2. The IgG Fc portions of the antibody construct were captured onto the biosensor and their interactions with Her2 measured with respect to the antigen association (*K*_on_) and dissociation rate (*K*_off_). The calculated equilibrium dissociation constants (*K*_D_) of the original pairing of Pertuzumab and Trastuzumab are 0.87 × 10^−9^ and 0.30 × 10^−9^ M respectively, which were similar to that previously reported (14, 15, 29).

**Figure 5:**
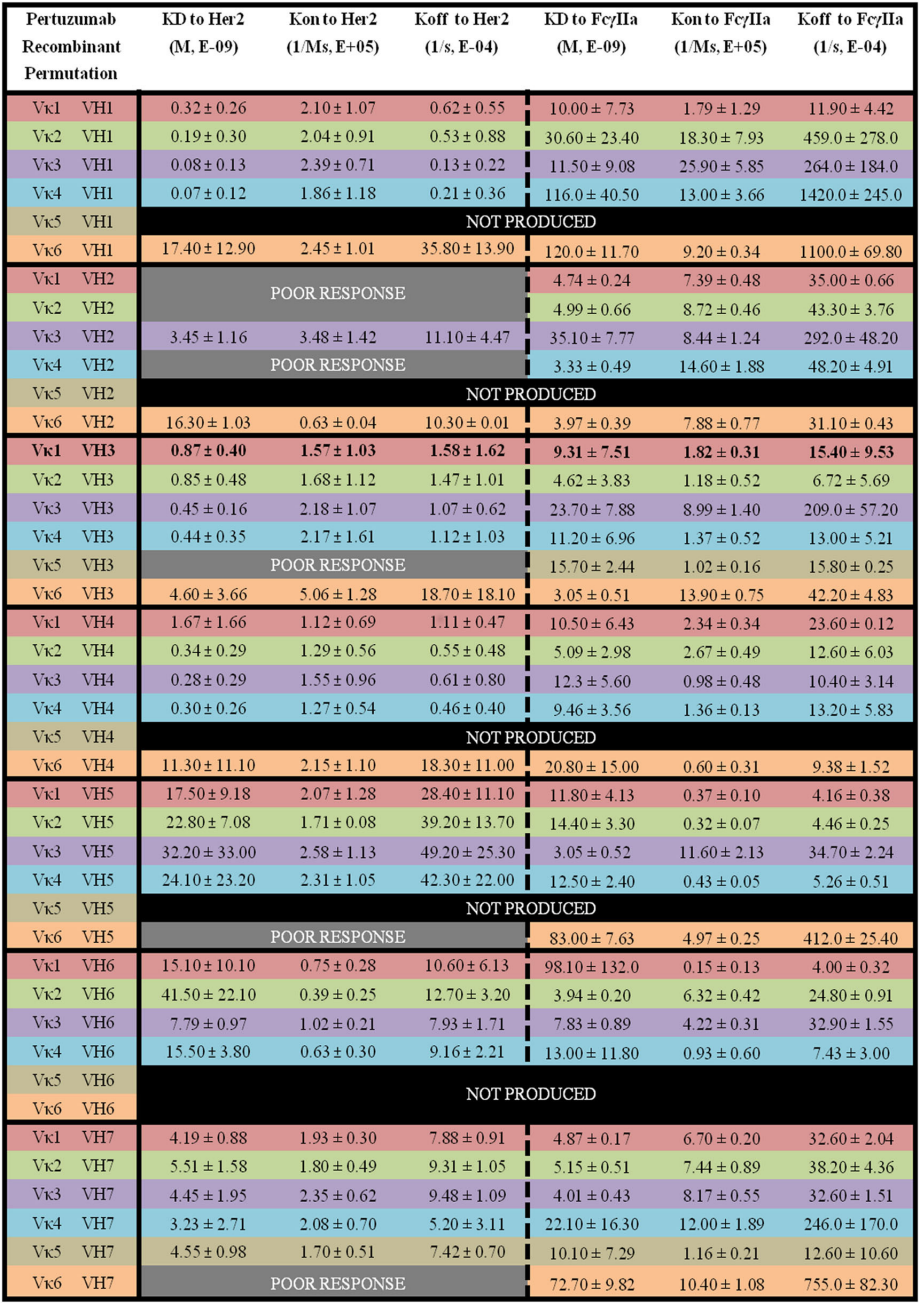
The binding kinetics of recombinant Pertuzumab to Her2 and FcγIIa. The recombinant antibody variants are arranged according to VH families and color coded for Vκ (Vκ1—red, Vκ2—green, Vκ3—purple, Vκ4—blue, Vκ5—brown, and Vκ6—orange) while the original pairing is bolded with *K*_D_, *K*_on_, and *K*_off_ measurements shown. “Poor response” indicates that the antibody combinations did not yield reliable *K*_on_ and *K*_off_ during measurements (in triplicates) as determined by the Octet software. “Not produced” indicates that there was no useable amount of the purified product despite several transfection attempts.

Among the Pertuzumab variants, Vκ4|VH1 (*K*_D_ = 0.07 ± 0.12 × 10^−9^ M) showed the lowest equilibrium dissociation constant (strongest binder) or *K*_D_ while Vκ2|VH6 (*K*_D_ = 41.50 ± 22.10 × 10^−9^ M) had the highest *K*_D_ (weakest binder). Vκ1|VH2, Vκ2|VH2, Vκ4|VH2, Vκ5|VH3, Vκ6|VH5, and Vκ6|VH7 gave poor responses indicating that they were not able to bind Her2 at detectable amounts, and most Vκ5 paired anti-bodies did not yield recombinant whole IgG1 at sufficient quantities for further analyses, with the exception when paired up with VH3 and 7. The trend for strong binding to Her2 based on low *K*_D_ for the Vκ families are as follows: Vκ3 > 4 > 2 > 1 > 6 > 5. Whereas for the VH families, they are: VH1 > 4 > 3 > 7 > 6 > 5 > 2. The Vκ FWRs did not seem to influence Her2 binding significantly. However, in our dataset, we found exceptions for the Vκ5 and 6 pairs that influenced irregularity within the same VH FWR family. Consistent with general antibody research, VH FWR families showed more effect on the Her2 binding measurements as observed in Figure 5.

**Figure 6:**
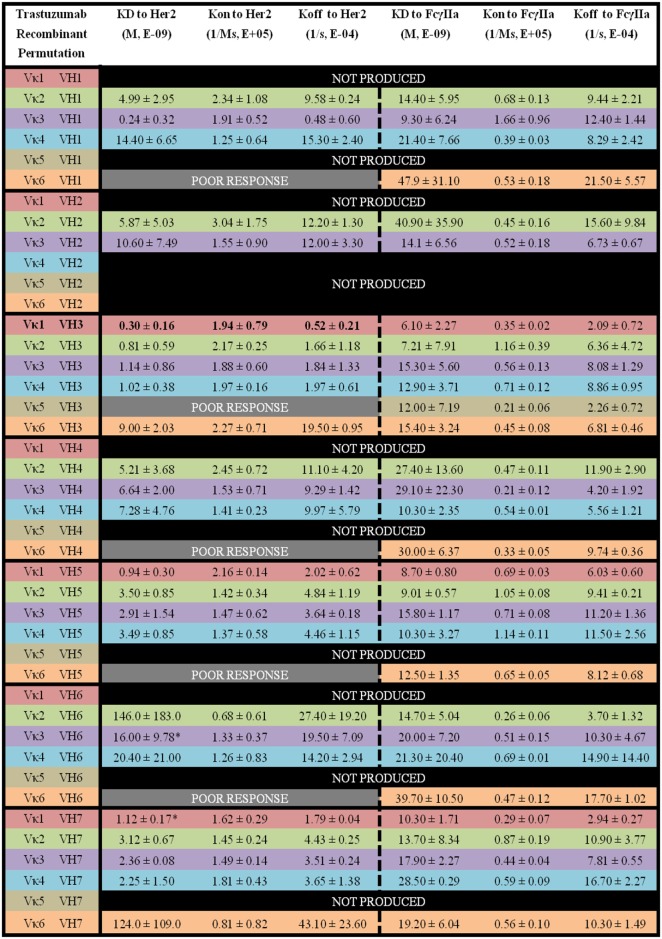
The binding kinetics of recombinant Trastuzumab to Her2 and FcγIIa. The recombinant antibody variants are arranged according to VH families and color coded for Vκ (Vκ1—red, Vκ2—green, Vκ3—purple, Vκ4—blue, Vκ5—brown, and Vκ6—orange) while the original pairing is bolded with *K*_D_, *K*_on_, and *K*_off_. “Poor response” indicates that the antibody combinations did not yield reliable *K*_on_ and *K*_off_ during measurements (in triplicates) as determined by the Octet software. “Not produced” indicates that there was no useable amount of the purified product despite several transfection attempts.

Within the Trastuzumab combinations, Vκ3|VH1 (*K*_D_ = 0.24 ± 0.32 × 10^−9^ M) had the lowest *K*_D_ while Vκ2|VH6 (*K*_D_ = 146.0 ± 183.0 × 10^−9^ M) showed the highest readings. Vκ6|VH1, Vκ5|VH3, Vκ6|VH4, Vκ6|VH5, and Vκ6|VH6 pairs gave poor responses for measurement. The trends of Her2 antigen binding for the Vκ families are as follows: Vκ1 > 3 > 4 > 2 > 6 > 5, while VH families are as follows: VH3 > 7 > 5 > 1 > 4 > 2 > 6. Similar to Pertuzumab constructs, Vκ FWRs did not exert major effects on Her2 binding, with the exception of Vκ6 that consistently had very high *K*_off_ regardless of the VH paired with. When paired with VH1, a greater variation in *K*_off_ was observed. VH FWRs also affected Her2 binding with respect to *K*_on_ and *K*_off_ for the Trastuzumab variants as shown in Figure 6.

### Effects of VL and VH FWR Families on Receptor Binding Kinetics to FcγIIA

Using the same 66 purified variants of whole Trastuzumab and Pertuzumab IgG1, we proceeded to measure the *K*_on_ and *K*_off_ as well as *K*_D_ of these whole IgG1 variants to FcγIIA receptor.

Of the Pertuzumab variants, there was a range of differences in *K*_D_. Both Vκ3|VH5 (*K*_D_ = 3.05 ± 0.52 × 10^−9^ M) and Vκ3|VH3 (*K*_D_ = 3.05 ± 0.51 × 10^−9^ M) pairs showed the lowest *K*_D_ (strongest binders) while the Vκ6|VH1 pair (*K*_D_ = 120.0 ± 11.70 × 10^−9^ M) had the highest *K*_D_ (weakest binder). There is no obvious trend although Vκ1, 2, and 3 had lower *K*_D_ values compared with Vκ4, 5, and 6 for Vκ families while VH1, 2, and 3 had lower *K*_D_ values compared with VH4, 5, 6, and 7. Interestingly, VH3 is the only family that showed an opposite trend (see Figure 5 for detailed readings).

For the Trastuzumab combinations, a smaller range of *K*_D_ was observed when compared with the Pertuzumab variants. The Vκ1|VH3 pair (*K*_D_ = 6.10 ± 2.27 × 10^−9^ M) had the lowest *K*_D_ while the Vκ2|VH2 pair (40.90 ± 35.90 × 10^−9^ M) had the highest *K*_D_. In contrast to Pertuzumab variants, Trastuzumab variants had no combinations that gave a poor response against FcγIIa binding. The trend of lowest to highest *K*_D_ for the Vκ families in *K*_D_ to FcγIIA binding are as follows: Vκ1 > 2 > 3 > 4 > 6 > 5, while that of the VH families are as follows: VH3 > 5 > 7 > 1 > 4 > 2 > 6 (Figure 6).

Based on both the results of the Pertuzumab and Trastuzumab variants in their binding measurements to the FcγIIA receptor, there were no clear major effects elicited by the FWRs, but rather by the CDRs. The only exception to this was for the Vκ5 family, which consistently gave low measurements. However, this same family also did not generally produce well nor did it give good binding measurements to Her2.

## Discussion

We sought out to investigate the effects of VL (specifically Vκ) and VH families on recombinant transient antibody production, antigen binding, and IgG receptor engagement, all three of which, particularly the latter two, are important elements to make successful biologics. While high yielding antibodies are desired for preliminary testing before selection for downstream processes such as permanent recombinant cell production, the therapeutic candidate must bind and flag the target antigen for immune effector cells *via* appropriate receptors. Given that the effects of VH and VL FWRs in the humanization grafting process remain to be fully characterized for the above effects on whole antibodies, we set out to do this using the well-studied Trastuzumab and Pertuzumab as models, especially given that they bind to the same antigen, albeit on different epitopes.

In agreement with a previous study (30), we found that the VH3 family pairs gave the highest production yields among the other VH family pairs. In the analysis of our panel of antibodies, there is general agreement with previous data that the light chain plays a role for good antibody production (31) and in antigen-binding measurements (32). While the VH family appears to play the major role to influence production rates, the Vκ family appears to fine tune both recombinant production and antigen-binding measurements. For example, an existing Vκ2|VH1 may not require switching to VH3 to boost production, but can be paired with another Vκ family FWR to improve transient production and binding kinetics. Furthermore in our panel, the Vκ3|VH1 pair was transiently produced 1-fold higher than the Vκ2|VH1 pair while retaining similar binding measurements, showing that antibody production rates are not necessarily correlated with Her2 and FcγIIA binding in our panel.

Additional reasons for light chain switching from Vκ2|VH1 to Vκ3|VH1 would be for purification, e.g., protein L, which is one of the most commonly used methods to purify recombinant mAbs and binds only to Vκ1, Vκ3, and Vκ4 families but not Vκ2 (10). Utilizing light chain targeted purifications would have value when purifying antibodies of constant region variants (15).

Despite having certain grafted chains, e.g., Vκ5 that were generally not well produced (with few exceptions) and had poor binding measurements to both Her2 and FcγIIA, it is possible that the grafting for Vκ5 required other deeper investigation, especially given that it is one of the least documented sequences in IMGT database at the point of writing.

In our antibody panel, we did not observe aggregation, though there were certain combinations such as Vκ5 in Pertuzumab recombinant variants and VH1, 2, and 6 in Trastuzumab recombinant variants that showed more free light chains being secreted (Figures S3 and S4 in Supplementary Material).

Nonetheless, the above results on production issues are based solely on small scale transient production and are not likely to be transferable to industrial permanent antibody production. For this reason, this research is focused on antigen and receptor binding.

We observed the VH and VL FWRs to affect Her2 binding measurements in our panel of both Trastuzumab and Pertuzumab whole IgG1 variants. We found the Vκ3|VH1 pairs of both Trastuzumab and Pertuzumab to exhibit the strongest binding (lowest *K*_D_ values) within each model pool when binding to Her2. Such observations point to the need to reconsider the usual selection of VH3 for therapeutic antibodies beyond only production considerations. This is demonstrated where despite showing that most Trastuzumab and Pertuzumab variants were capable of binding Her2, there were certain VH and VL families that showed compromised binding measurements when compared with the original Trastuzumab and Pertuzumab IgG1 pairs.

From our panel, we observed that the VH and VL FWRs affected the binding of the whole IgG1 variants to FcγIIA. This observation raises many questions to the pipeline of therapeutic antibody development, and general antibody research and characterization that do not involve whole antibodies (e.g. affinity maturation using display methods) as they neglect the engagement of FcR. While such methods may certainly yield supe-rior antigen-binding antibodies, they may not yield a successful therapeutic if the activity of the biologic was to be reliant on antibody-dependent cell cytotoxicity *via* FcRs. In fact, the original Trastuzumab, which was very successful in clinical treatment, had the best binding to FcγIIA among the Trastuzumab variants, whereas for the Pertuzumab variants, Vκ6|VH3 was the best binding pair within its own variant pool. As shown in our panel, there are variants from the Pertuzumab pool (e.g., Vκ4|VH1 and Vκ6|VH1 pairs) that despite showing capabilities to bind Her2 (although Vκ6|VH1 showed weaker binding), had compromised FcγIIA binding. At the same time, our findings propose a solution to such problems where changing the Vκ partner can be performed to restore FcγIIA binding without compromising Her2 binding significantly. Since general FcR engagement is important for clinical efficacy (33) in immunotherapy, a therapeutic antibody that has poor FcR engagement is likely to show poorer clinical efficacy compared with other VH–VL variants that bind better.

Comparing the Pertuzumab and Trastuzumab pools which differ only in CDRs, we found the CDRs to impact transient production, Her2 and FcγIIA binding. Considering our entire data, there are clear combinatorial effects for both FWRs and CDRs on these parameters. Since current technology does not include effective direct CDR engineering, our findings propose that the simpler option is to optimize therapeutic antibodies *via* the VH and VL FWRs. While there have been mapped effects within frameworks, e.g., formation of disulfide bond (34), glycosylation (35), Fc mutation for FcR binding (36), half-lives (37), there is a need to consider that various antibody elements do influence one another. Together with our other work where the swapping of human Ig isotypes can affect antigen binding (15), we propose a rethink of the commonly accepted assumption that only the Ig Fc is involved in FcR engagement (e.g., IgGs to FcγR, IgE to FcεR, and IgAs to FcαR), and that only the CDRs are solely responsible for antigen binding. This calls for a rethink of general antibody research and therapeutic development pipelines where research not involving whole antibodies or FcR analysis may require an overhaul in approach. Given that our results here show that despite playing a major role, the CDRs alone certainly do not solely confer the necessary antigen or FcR binding properties, this poses a question to whether intellectual protection of antibody CDRs alone sufficiently reflects a whole therapeutic antibody. This is especially so, given that there is no one template that provides the best optimized antibody despite some trends, and that every therapeutic antibody may require individualized optimizations.

In conclusion, our study proposes a list of VH and VL FWRs (Figure 1) that can be used for antibody grafting and is the first to incorporate receptor binding kinetics for a more comprehensive characterization of antibodies from the typical assessment of production and antigen binding. In this process, we were able to map the influence of the VH and VL family pairings for Pertuzumab and Trastuzumab. While the VH FWR families play a major role in production and both antigen and FcR binding, the VL FWR families can be used to fine tune these parameters. And these findings are certainly relevant to antibody engineers that optimize therapeutic antibodies.

## Author Contributions

WLL, WHL, and SG analyzed the results. WLL, WHL, JJP, and JY performed the wet lab experiments. WLL, WHL, and SG wrote the manuscript. DL approved and revised the manuscript. SG designed and supervised all aspects of the study. All the authors read and approved the manuscript.

## Conflict of Interest Statement

The authors declare that the research was conducted in the absence of any commercial or financial relationships that could be construed as a potential conflict of interest. The reviewer ZA and handling editor declared their shared affiliation.
