# Supplementary material for: Effect of VH–VL Families in Pertuzumab and Trastuzumab Recombinant Production, Her2 and FcγIIA Binding
Source: Front Immunol. 2018 Mar 12;9:469. doi: 10.3389/fimmu.2018.00469 (PMC5857972; doi:10.3389/fimmu.2018.00469)
Supplement: Supplementary file 1 [file Data_Sheet_1.DOCX]

Supplementary Material

**Effect of VH-VL families in Pertuzumab and Trastuzumab recombinant production, Her2 and FcγIIA-binding**

**Wei-Li Ling^1^, Wai-Heng Lua^1^, Jun-Jie Poh^1^, Joshua Yi Yeo^1^, David Philip Lane^2^, Samuel Ken-En Gan^1, 2,*^**

***Correspondence:** Samuel Ken-En Gan: samuelg@bii.a-star.edu.sg

# Supplementary Figures

**
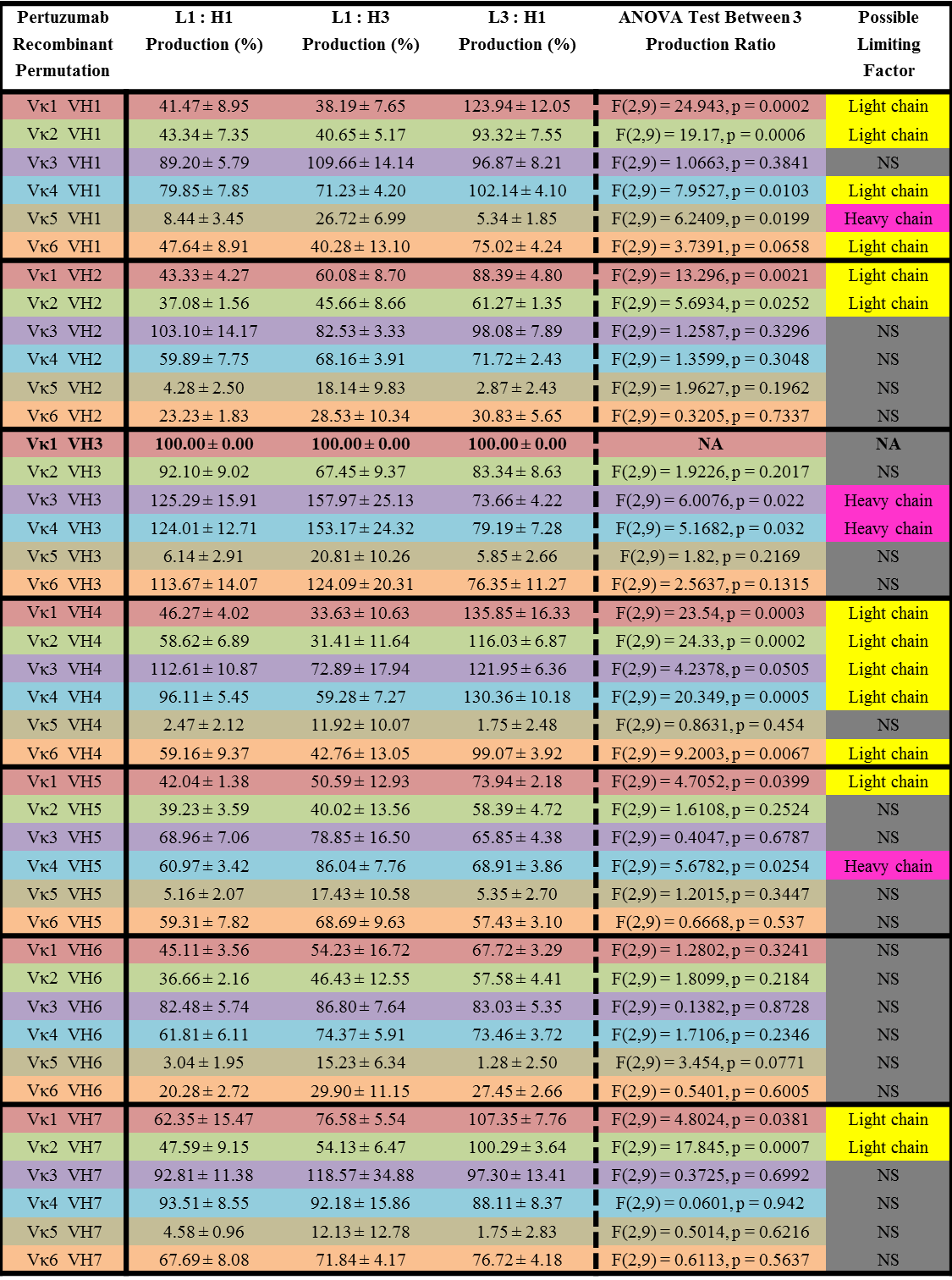
**

**Supplementary Figure 1.** Production levels in percentages of recombinant Pertuzumab variants are shown in percentages with respect to the original Vκ3|VH1 pair. The antibody variants are arranged according to VH families and colour coded for Vκ (Vκ1 - Red, Vκ2 - Green, Vκ3 - Purple, Vκ4 - Blue, Vκ5 - Brown and Vκ6 - Orange) with light chain : heavy chain plasmid in ratios of 1:1, 1:3, 3:1. Limiting chains were determined using ANOVA test at p < 0.05.


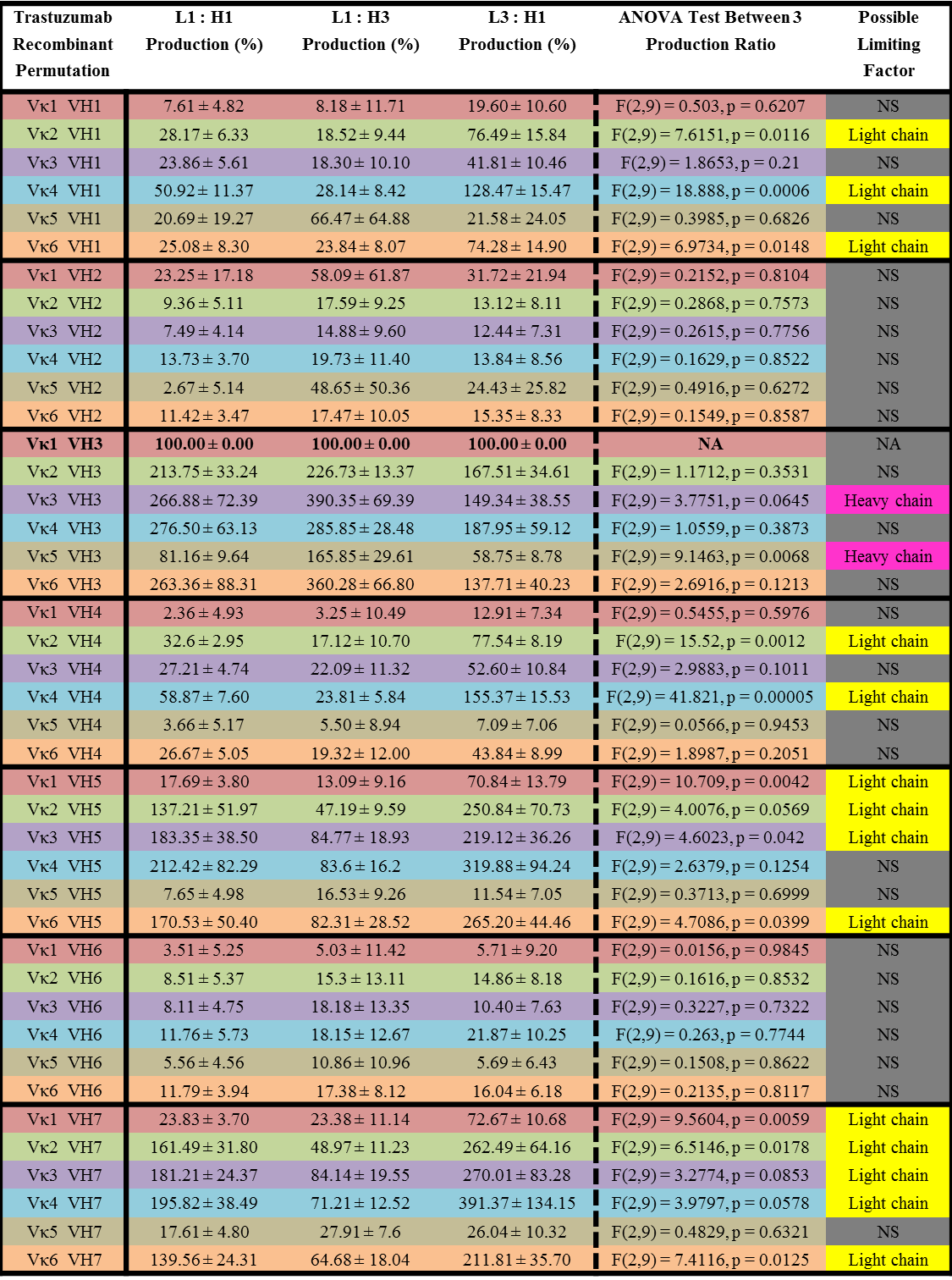


**Supplementary Figure 2.** Production levels in percentages of recombinant Trastuzumab variants are shown are shown in percentages with respect to the original Vκ3|VH1 pair. The antibody variants are arranged according to VH families and colour coded for Vκ (Vκ1 - Red, Vκ2 - Green, Vκ3 - Purple, Vκ4 - Blue, Vκ5 - Brown and Vκ6 - Orange) with light chain : heavy chain plasmid in ratios of 1:1, 1:3, 3:1. Limiting chains were determined using ANOVA test at p < 0.05.


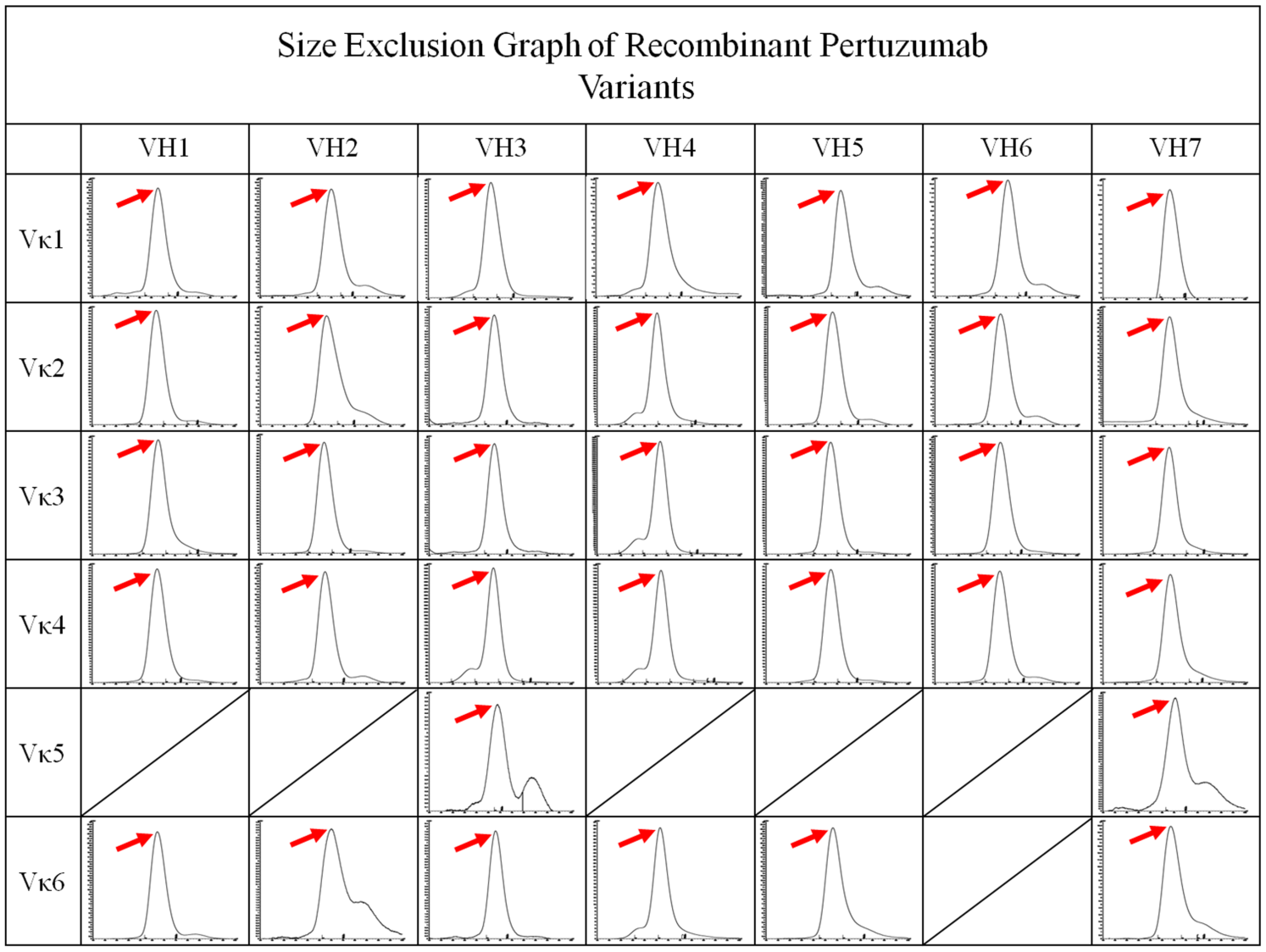


**Supplementary Figure 3.** Size exclusion chromatograms of the recombinant Pertuzumab variants. Antibodies that were produced in detectable amounts were subjected to size exclusion chromatography after Protein G affinity purifications to obtain pure monomeric fraction peaks as indicated by the red arrow. The monomeric fractions were determined based on the elution volume (at ~70 ml) as was previously calibrated (Lua et al., 2018, Su et al., 2017).


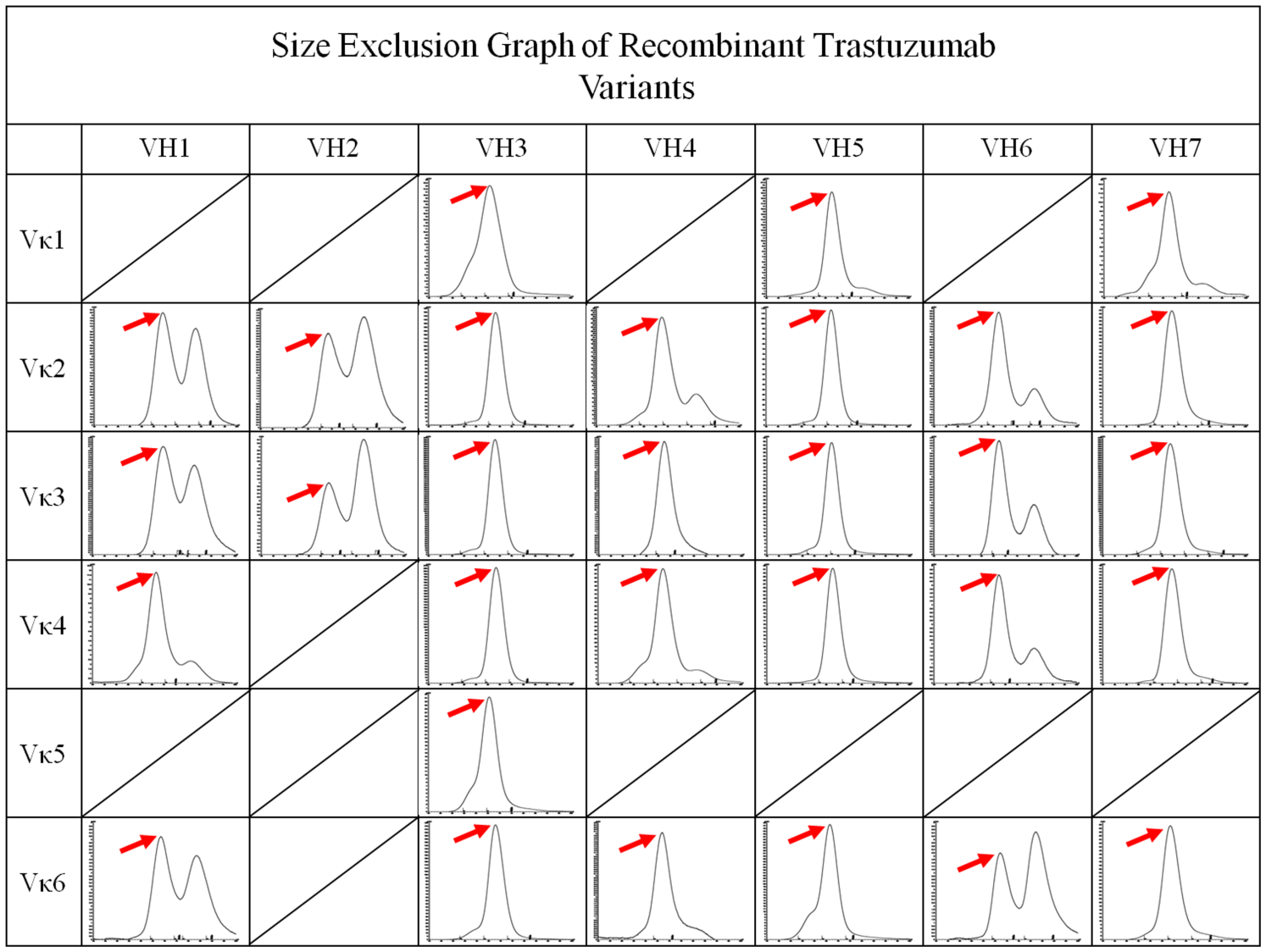


**Supplementary Figure 4.** Size exclusion chromatograms of the recombinant Trastuzumab variants. Antibodies that were produced in detectable amounts were subjected to size exclusion chromatography after Protein G affinity purifications to obtain pure monomeric fraction peaks as indicated by the red arrow. The monomeric fractions were determined based on the elution volume (at ~70 ml) as was previously calibrated (Lua et al., 2018, Su et al., 2017).

# Supplementary Data

>Trastuzumab VK2

DIVMTQSPLSLPVTPGEPASISCRASQDVNTAVAWYLQKPGQSPQLLIYSASFLYSGVPDRFSGSGSGTDFTLKISRVEAEDVGVYYCQQHYTTPPTFGQGTKVEIK

>Pertuzumab VK2

DIVMTQSPLSLPVTPGEPASISCKASQDVSIGVAWYLQKPGQSPQLLIYSASYRYSGVPDRFSGSGSGTDFTLKISRVEAEDVGVYYCQQYYIYPYTFGQGTKVEIK

>Trastuzumab VK3

EIVLTQSPATLSLSPGERATLSCRASQDVNTAVAWYQQKPGQAPRLLIYSASFLYSGVPARFSGSGSGTDFTLTISSLEPEDFAVYYCQQHYTTPPTFGQGTKVEIK

>Pertuzumab VK3

EIVLTQSPATLSLSPGERATLSCKASQDVSIGVAWYQQKPGQAPRLLIYSASYRYSGVPARFSGSGSGTDFTLTISSLEPEDFAVYYCQQYYIYPYTFGQGTKVEIK

>Trastuzumab VK4

DIVMTQSPDSLAVSLGERATINCRASQDVNTAVAWYQQKPGQPPKLLIYSASFLYSGVPDRFSGSGSGTDFTLTISSLQAEDVAVYYC**QQ**HYTTPPTFGQGTKVEIK

>Pertuzumab VK4

DIVMTQSPDSLAVSLGERATINCKASQDVSIGVAWYQQKPGQPPKLLIYSASYRYSGVPDRFSGSGSGTDFTLTISSLQAEDVAVYYC**QQ**YYIYPYTFGQGTKVEIK

>Trastuzumab VK5

ETTLTQSPAFMSATPGDKVNISCRASQDVNTAVAWYQQKPGEAAIFIIQSASFLYPGIPPRFSGSGYGTDFTLTINNIESEDAAYYFCQQHYTTPPTFGQGTKVEIK

>Pertuzumab VK5

ETTLTQSPAFMSATPGDKVNISCKASQDVSIGVAWYQQKPGEAAIFIIQSASYRYPGIPPRFSGSGYGTDFTLTINNIESEDAAYYFCQQYYIYPYTFGQGTKVEIK

>Trastuzumab VK6

EIVLTQSPDFQSVTPKEKVTITCRASQDVNTAVAWYQQKPDQSPKLLIKSASFLYSGVPSRFSGSGSGTDFTLTINSLEAEDAATYYCQQHYTTPPTFGQGTKVEIK

>Pertuzumab VK6

EIVLTQSPDFQSVTPKEKVTITCKASQDVSIGVAWYQQKPDQSPKLLIKSASYRYSGVPSRFSGSGSGTDFTLTINSLEAEDAATYYCQQYYIYPYTFGQGTKVEIK

>Trastuzumab VH1

QVQLVQSGVEVKKPGASVKVSCKASGFNIKDTYIHWVRQAPGQGLEWMARIYPTNGYTRYADSVKGRVTLTTDSSTTTAYMELKSLQFDDTAVYYCSRWGGDGFYAMDYWGQGTLVTVSS

>Pertuzumab VH1

QVQLVQSGVEVKKPGASVKVSCKASGFTFTDYTMDWVRQAPGQGLEWMADVNPNSGGSIYNQRFKGRVTLTTDSSTTTAYMELKSLQFDDTAVYYCARNLGPSFYFDYWGQGTLVTVSS

>Trastuzumab VH2

QVTLRESGPALVKPTQTLTLTCTFSGFNIKDTYIHWIRQPPGKALEWLARIYPTNGYTRYADSVKGRLTISKDTSKNQVVLKVTNMDPADTATYYCSRWGGDGFYAMDYWGQGTLVTVSS

>Pertuzumab VH2

QVTLRESGPALVKPTQTLTLTCTFSGFTFTDYTMDWIRQPPGKALEWLADVNPNSGGSIYNQRFKGRLTISKDTSKNQVVLKVTNMDPADTATYYCARNLGPSFYFDYWGQGTLVTVSS

>Trastuzumab VH4

QVQLKQSGPGLVQPSQSLSITCTVSGFNIKDTYIHWVRQPPGRGLEWIARIYPTNGYTRYADSVKGRVTMLVDTSKNQFSLRLSSVTAADTAVYYCSRWGGDGFYAMDYWGQGTLVTVSS

>Pertuzumab VH4

QVQLKQSGPGLVQPSQSLSITCTVSGFTFTDYTMDWVRQPPGRGLEWIADVNPNSGGSIYNQRFKGRVTMLVDTSKNQFSLRLSSVTAADTAVYYCARNLGPSFYFDYWGQGTLVTVSS

>Trastuzumab VH5

EVQLVQSGAEVKKPGESLRISCKGSGFNIKDTYIHWVRQMPGKGLEWMARIYPTNGYTRYADSVKGHVTISADKSISTAYLQWSSLKASDTAMYYCSRWGGDGFYAMDYWGQGTLVTVSS

>Pertuzumab VH5

EVQLVQSGAEVKKPGESLRISCKGSGFTFTDYTMDWVRQMPGKGLEWMADVNPNSGGSIYNQRFKGHVTISADKSISTAYLQWSSLKASDTAMYYCARNLGPSFYFDYWGQGTLVTVSS

>Trastuzumab VH6

QVQLQQSGPGLVKPSQTLSLTCAISGFNIKDTYIHWIRQSPSRGLEWLARIYPTNGYTRYADSVKGRITINPDTSKNQFSLQLNSVTPEDTAVYYCSRWGGDGFYAMDYWGQGTLVTVSS

>Pertuzumab VH6

QVQLQQSGPGLVKPSQTLSLTCAISGFTFTDYTMDWIRQSPSRGLEWLADVNPNSGGSIYNQRFKGRITINPDTSKNQFSLQLNSVTPEDTAVYYCARNLGPSFYFDYWGQGTLVTVSS

>Trastuzumab VH7

EVQLVESGGGLVQPGGSLRLSCAASGFNIKDTYIHWVRQAPGKGLEWVARIYPTNGYTRYADSVKGRFTFSLDTSKSTAYLQMNSLRAEDTAVYYCSRWGGDGFYAMDYWGQGTLVTVSS

>Pertuzumab VH7

EVQLVESGGGLVQPGGSLRLSCAASGFTFTDYTMDWVRQAPGKGLEWVADVNPNSGGSIYNQRFKGRFTFSLDTSKSTAYLQMNSLRAEDTAVYYCARNLGPSFYFDYWGQGTLVTVSS

**Supplementary Data 1**. List of grafted antibody sequences according to their respective light and heavy chain FWRs family with Pertuzumab and Trastuzumab CDRs used in this study. FWRs are highlighted in green, Pertuzumab CDRs are highlighted in cyan, Trastuzumab CDRs are highlighted in yellow and joining chain are highlighted in purple.

**Vκ1**

>Bevacizumab Light chain

DIQMTQSPSSLSASVGDRVTITCSASQDISNYLNWYQQKPGKAPKVLIYFTSSLHSGVPSRFSGSGSGTDFTLTISSLQPEDFATYYCQQYSTVPWTFGQGTKVEIKR

>**Trastuzumab** Light chain

DIQMTQSPSSLSASVGDRVTITCRASQDVNTAVAWYQQKPGKAPKLLIYSASFLYSGVPSRFSGSRSGTDFTLTISSLQPEDFATYYCQQHYTTPPTFGQGTKVEIKR

>**Alemtuzumab** Light Chain

DIQMTQSPSSLSASVGDRVTITCKASQNIDKYLNWYQQKPGKAPKLLIYNTNNLQTGVPSRFSGSGSGTDFTFTISSLQPEDIATYYCLQHISRPRTFGQGTKVEIKR

>Omalizumab Light chain

DIQLTQSPSSLSASVGDRVTITCRASQSVDYDGDSYMNWYQQKPGKAPKLLIYAASYLESGVPSRFSGSGSGTDFTLTISSLQPEDFATYYCQQSHEDPYTFGQGTKVEIKR

>**Efalizumab** Light Chain

DIQMTQSPSSLSASVGDRVTITCRASKTISKYLAWXQQKPGKAPKLLIYSGSTLQSGVPSRFSGSGSGTDFTLTISSLQPEDFATYYCQQHNEYPLTFGQGTKVEIKR

>Ranibizumab Light Chain

DIQLTQSPSSLSASVGDRVTITCSASQDISNYLNWYQQKPGKAPKVLIYFTSSLHSGVPSRFSGSGSGTDFTLTISSLQPEDFATYYCQQYSTVPWTFGQGTKVEIKR

>**Daclizumab** Light Chain

DIQMTQSPSTLSASVGDRVTITCSASSSISYMHWYQQKPGKAPKLLIYTTSNLASGVPARFSGSGSGTEFTLTISSLQPDDFATYYCHQRSTYPLTFGSGTKVEVKR

>**Certolizumab pegol** Light chain

DIQMTQSPSSLSASVGDRVTITCKASQNVGTNVAWYQQKPGKAPKALIYSASFLYSGVPYRFSGSGSGTDFTLTISSLQPEDFATYYCQQYNIYPLTFGQGTKVEIKR

>**Pexelizumab** Light Chain

DIQMTQSPSLSASVGDRVTITCGASENIYGALNWYQRKPGKAPKLLIYGATNLADGVPSRFSGSGSGTDYTLTISSLQPEDFATYYCQNVLNTPLTFGQGTKVEIK

>Oportuzumab monatox Light Chain

DIQMTQSPSSLSASVGDRVTITCRSTKSLLHSNGITYLYWYQQKPGKAPKLLIYQMSNLASGVPSRFSSSGSGTDFTLTISSLQPEDFATYYCAQNLEIPRTFGQGTKVELKR

>**Natalizumab Light Chain**

DIQMTQSPSSLSASVGDRVTITCKTSQDINKYMAWYQQTPGKAPRLLIHYTSALQPGIPSRFSGSGSGRDYTFTISSLQPEDIATYYCLQYDNLWTFGQGTKVEIKR

>Pertuzumab Light chain

DIQMTQSPSSLSASVGDRVTITCKASQDVSIGVAWYQQKPGKAPKLLIYSASYRYTGVPSRFSGSGSGTDFTLTISSLQPEDFATYYCQQYYIYPYTFGQGTKVEIKR

**Vκ2**

>**Vedolizumab** Light Chain

DVVMTQSPLSLPVTPGEPASISCRSSQSLAKSYGNTYLSWYLQKPGQSPQLLIYGISNRFSGVPDRFSGSGSGTDFTLKISRVEAEDVGVYYCLQGTHQPYTFGQGTKVEIKR

**Vκ3**

>**Gemtuzumab ozogamicin** Light Chain

QIVLTQSPAIMSASPGEKVTITCSASSSISYMHWFQQKPGTSPKLWIYTTSNLASGVPARFSGSGSGTSYSLTISRMEAEDAATYYCHQRSTYPLTFGSGTKLELK

>Cetuximab Light chain

DILLTQSPVILSVSPGERVSFSCRASQSIGTNIHWYQQRTNGSPRLLIKYASESISGIPSRFSGSGSGTDFTLSINSVESEDIADYYCQQNNNWPTTFGAGTKLELKR

>Matuzumab Light chain

DIQMTQSPSSLSASVGDRVTITCSASSSVTYMYWYQQKPGKAPKLLIYDTSNLASGVPSRFSGSGSGTDYTFTISSLQPEDIATYYCQQWSSHIFTFGQGTKVEIKR

>**Pembrolizumab** Light Chain

EIVLTQSPATLSLSPGERATLSCRASKGVSTSGYSYLHWYQQKPGQAPRLLIYLASYLESGVPARFSGSGSGTDFTLTISSLEPEDFAVYYCQHSRDLPLTFGGGTKVEIKR

**Supplementary Data 2**. List of commercial antibody sequences group according to their respective light chain family after subjecting to IMGT V-QUEST for variable family identification. There was no commercial antibody of Vκ4, 5 and 6 families at the point of writing.

**VH1**

>**Gemtuzumab ozogamicin** Heavy Chain

QVQLQQSGAELAKPGASVKMSCKASGYTFTSYRMHWVKQRPGQGLEWIGYINPSTGYTEYNQKFKDKATLTADKSSSTAYMQLSSLTFEDSAVYYCARGGGVFDYWGQGTTLTVSS

>**Daclizumab** Heavy Chain

QVQLVQSGAEVKKPGSSVKVSCKASGYTFTSYRMHWVRQAPGQGLEWIGYINPSTGYTEYNQKFKDKATITADESTNTAYMELSSLRSEDTAVYYCARGGGVFDYWGQGTTLTVSS

>**Matuzumab Heavy Chain**

QVQLVQSGAEVKKPGASVKVSCKASGYTFTSHWMHWVRQAPGQGLEWIGEFNPSNGRTNYNEKFKSKATMTVDTSTNTAYMELSSLRSEDTAVYYCASRDYDYDGRYFDYWGQGTLVTVSS

>**Pexelizumab** Heavy Chain

QVQLVQSGAEVKKPGASVKVSCKASGYIFSNYWIQWVRQAPGQGLEWMGEILPGSGSTEYAQKFQGRVTMTADTSTSTAYMELSSLRSEDTAVYYCARYFFGSSPNWYFVWGQGT

>**Vedolizumab** Heavy Chain

QVQLVQSGAEVKKPGASVKVSCKGSGYTFTSYWMHWVRQAPGQRLEWIGEIDPSESNTNYNQKFKGRVTLTVDISASTAYMELSSLRSEDTAVYYCARGGYDGWDYAIDYWGQGTLVTVSS

>**Pembrolizumab** Heavy Chain

QVQLVQSGVEVKKPGASVKVSCKASGYTFTNYYMYWVRQAPGQGLEWMGGINPSNGGTNFNEKFKNRVTLTTDSSTTTAYMELKSLQFDDTAVYYCARRDYRFDMGFDYWGQGTTVTVSS

>**Natalizumab Heavy Chain**

QVQLVQSGAEVKKPGASVKVSCKASGFNIKDTYIHWVRQAPGQRLEWMGRIDPANGYTKYDPKFQGRVTITADTSASTAYMELSSLRSEDEAVYYCAREGYYGNYGVYAMDYWGQGTLVTVSS

**VH2**

>**Palivizumab Heavy** Chain

QVTLRESGPALVKPTQTLTLTCTFSGFSLSTSGMSVGWIRQPPGKALEWLADIWWDDKKDYNPSLKSRLTISKDTSKNQVVLKVTNMDPADTATYYCARSMITNWYFDVWGAGTTVTVSS

**VH3**

>Omalizumab Heavy chain

EVQLVESGGGLVQPGGSLRLSCAVSGYSITSGYSWNWIRQAPGKGLEWVASITYDGSTNYADSVKGRFTISRDDSKNTFYLQMNSLRAEDTAVYYCARGSHYFGHWHFAVWGQGTLVTVSS

>**Trastuzumab** Heavy chain

EVQLVESGGGLVQPGGSLRLSCAASGFNIKDTYIHWVRQAPGKGLEWVARIYPTNGYTRYADSVKGRFTISADTSKNTAYLQMNSLRAEDTAVYYCSRWGGDGFYAMDYWGQGTLVTVSS

>**Efalizumab** Heavy Chain

EVQLVESGGGLVQPGGSLRLSCAASGYSFTGHWMNWVRQAPGKGLEWVGIMIHPSDSETRYNQKFKDIRFTISVDKSKNTLYLQMNSLRAEDTAVYYCARIGIYFYGTTYFDYIWGQGTLVTVSS

>**Certolizumab pegol** Heavy chain

EVQLVESGGGLVQPGGSLRLSCAASGYVFTDYGMNWVRQAPGKGLEWMGWINTYIGEPIYADSVKGRFTFSLDTSKSTAYLQMNSLRAEDTAVYYCARGYRSYAMDYWGQGTLVTVSS

>Pertuzumab Heavy chain

EVQLVESGGGLVQPGGSLRLSCAASGFTFTDYTMDWVRQAPGKGLEWVADVNPNSGGSIYNQRFKGRFTLSVDRSKNTLYLQMNSLRAEDTAVYYCARNLGPSFYFDYWGQGTLVTVSS

**VH4**

>**Alemtuzumab** Heavy Chain (VH4)

QVQLQESGPGLVRPSQTLSLTCTVSGFTFTDFYMNWVRQPPGRGLEWIGFIRDKAKGYTTEYNPSVKGRVTMLVDTSKNQFSLRLSSVTAADTAVYYCAREGHTAAPFDYWGQGSLVTVSS

>Cetuximab Heavy chain (VH4)

QVQLKQSGPGLVQPSQSLSITCTVSGFSLTNYGVHWVRQSPGKGLEWLGVIWSGGNTDYNTPFTSRLSINKDNSKSQVFFKMNSLQSNDTAIYYCARALTYYDYEFAYWGQGTLVTVSA

**VH7**

>Ranibizumab Heavy Chain (VH7)

EVQLVESGGGLVQPGGSLRLSCAASGYDFTHYGMNWVRQAPGKGLEWVGWINTYTGEPTYAADFKRRFTFSLDTSKSTAYLQMNSLRAEDTAVYYCAKYPYYYGTSHWYFDVWGQGTLVTVSS

>Bevacizumab Heavy chain (VH7)

EVQLVESGGGLVQPGGSLRLSCAASGYTFTNYGMNWVRQAPGKGLEWVGWINTYTGEPTYAADFKRRFTFSLDTSKSTAYLQMNSLRAEDTAVYYCAKYPHYYGSSHWYFDVWGQGTLVTVSS

>Oportuzumab monatox Heavy Chain (VH7)

VKQAPGKGLEWMGWINTYTGESTYADSFKGRFTFSLDTSASAAYLQINSLRAEDTAVYYCARFAIKGDYWGQGTLLTVSS

**Supplementary Data 3**. List of commercial antibody sequences group according to their respective heavy chain family after subjecting to IMGT V-QUEST for variable family identification. There was no commercial antibody of VH5 and 6 families at the point of writing.

**Vκ CDR Determination**

Bevacizumab DIQMTQSPSSLSASVGDRVTITCSASQDI-----SNYLNWYQQKPGKAPKVLIYFTSSLH

Ranibizumab DIQLTQSPSSLSASVGDRVTITCSASQDI-----SNYLNWYQQKPGKAPKVLIYFTSSLH

Trastuzumab DIQMTQSPSSLSASVGDRVTITCRASQDV-----NTAVAWYQQKPGKAPKLLIYSASFLY

Certolizumab DIQMTQSPSSLSASVGDRVTITCKASQNV-----GTNVAWYQQKPGKAPKALIYSASFLY

Pertuzumab DIQMTQSPSSLSASVGDRVTITCKASQDV-----SIGVAWYQQKPGKAPKLLIYSASYRY

Efalizumab DIQMTQSPSSLSASVGDRVTITCRASKTI-----SKYLAWXQQKPGKAPKLLIYSGSTLQ

Omalizumab DIQLTQSPSSLSASVGDRVTITCRASQSVDY-DGDSYMNWYQQKPGKAPKLLIYAASYLE

Alemtuzumab DIQMTQSPSSLSASVGDRVTITCKASQNI-----DKYLNWYQQKPGKAPKLLIYNTNNLQ

Daclizumab DIQMTQSPSTLSASVGDRVTITCSASSSI------SYMHWYQQKPGKAPKLLIYTTSNLA

Oportuzumab DIQMTQSPSSLSASVGDRVTITCRSTKSLLHSNGITYLYWYQQKPGKAPKLLIYQMSNLA

Natalizumab DIQMTQSPSSLSASVGDRVTITCKTSQDI-----NKYMAWYQQTPGKAPRLLIHYTSALQ

Pexelizumab DIQMTQSPS-LSASVGDRVTITCGASENI-----YGALNWYQRKPGKAPKLLIYGATNLA

***:***** ************* ::. : : * *:.*****: **: .

Bevacizumab SGVPSRFSGSGSGTDFTLTISSLQPEDFATYYC**QQ**YSTVPWTFGQGTKVEIKR

Ranibizumab SGVPSRFSGSGSGTDFTLTISSLQPEDFATYYCQQYSTVPWTFGQGTKVEIKR

Trastuzumab SGVPSRFSGSRSGTDFTLTISSLQPEDFATYYCQQHYTTPPTFGQGTKVEIKR

Certolizumab SGVPYRFSGSGSGTDFTLTISSLQPEDFATYYCQQYNIYPLTFGQGTKVEIKR

Pertuzumab TGVPSRFSGSGSGTDFTLTISSLQPEDFATYYCQQYYIYPYTFGQGTKVEIKR

Efalizumab SGVPSRFSGSGSGTDFTLTISSLQPEDFATYYCQQHNEYPLTFGQGTKVEIKR

Omalizumab SGVPSRFSGSGSGTDFTLTISSLQPEDFATYYCQQSHEDPYTFGQGTKVEIKR

Alemtuzumab TGVPSRFSGSGSGTDFTFTISSLQPEDIATYYCLQHISRPRTFGQGTKVEIKR

Daclizumab SGVPARFSGSGSGTEFTLTISSLQPDDFATYYCHQRSTYPLTFGSGTKVEVKR

Oportuzumab SGVPSRFSSSGSGTDFTLTISSLQPEDFATYYCAQNLEIPRTFGQGTKVELKR

Natalizumab PGIPSRFSGSGSGRDYTFTISSLQPEDIATYYCLQYDNL-WTFGQGTKVEIKR

Pexelizumab DGVPSRFSGSGSGTDYTLTISSLQPEDFATYYCQNVLNTPLTFGQGTKVEIK-

*:* ***.* ** ::*:*******:*:***** : ***.*****:*

**Supplementary Data 4**. Multiple sequence alignment of commercial light chain family for CDRs determination of Pertuzumab and Trastuzumab. Light chain of same family (Vκ1) with Pertuzumab and Trastuzumab were used for comparison. We determine that the most contrasting region belongs to CDR regions highlighted in red while Pertuzumab CDRs are highlighted in cyan and Trastuzumab CDRs are highlighted in yellow.

**Vκ2 Framework Determination**

Vedolizumab DVVMTQSPLSLPVTPGEPASISCRSSQSLAKS-YGNTYLSWYLQKPGQSPQLLIYGISNR

X12684|IGKV2-24 DIVMTQTPLSSPVTLGQPASISCRSSQSLVHS-DGNTYLSWLQQRPGQPPRLLIYKISNR

X63403|IGKV2-30 DVVMTQSPLSLPVTLGQPASISCRSSQSLVYS-DGNTYLNWFQQRPGQSPRRLIYKVSNR

FM164408|IGKV2- DVVMTQSPLSLPVTLGQPASISCRSSQSLVHS-DGNTYLNWFQQRPGQSPRRLIYKVSNR

X63402|IGKV2D-3 DVVMTQSPLSLPVTLGQPASISCRSSQSLVYS-DGNTYLNWFQQRPGQSPRRLIYKVSNW

X63397|IGKV2-28 DIVMTQSPLSLPVTPGEPASISCRSSQSLLHS-NGYNYLDWYLQKPGQSPQLLIYLGSNR

X12691|IGKV2D-2 DIVMTQSPLSLPVTPGEPASISCRSSQSLLHS-NGYNYLDWYLQKPGQSPQLLIYLGSNR

X63396|IGKV2-29 DIVMTQTPLSLSVTPGQPASISCKSSQSLLHS-DGKTYLYWYLQKPGQSPQLLIYEVSSR

U41645|IGKV2-29 DIVMTQTPLSLSVTPGQPASISCKSSQSLLHS-DGKTYLYWYLQKPGQSPQLLIYEVSSR

AJ783437|IGKV2- DIVMTQTPLSLSVTPGQPASISCKSSQSLLHS-DGKTYLYWYLQKPGQSPQLLIYEVSSR

M31952|IGKV2D-2 DIVMTQTPLSLSVTPGQPASISCKSSQSLLHS-DGKTYLYWYLQKPGQPPQLLIYEVSNR

U41644|IGKV2D-2 DIVMTQTPLSLSVTPGQPASISCKSSQSLLHS-DGKTYLYWYLQKPGQSPQLLIYEVSNR

X59314|IGKV2-40 DIVMTQTPLSLPVTPGEPASISCRSSQSLLDSDDGNTYLDWYLQKPGQSPQLLIYTLSYR

X59311|IGKV2D-4 DIVMTQTPLSLPVTPGEPASISCRSSQSLLDSDDGNTYLDWYLQKPGQSPQLLIYTLSYR

AP001216|IGKV2D EIVMTQTPLSLSITPGEQASISCRSSQSLLHS-DGYTYLYWFLQKARPVSTLLIYEVSNR

KM455565|IGKV2D EIVMTQTPLSLSITPGEQASMSCRSSQSLLHS-DGYTYLYWFLQKARPVSTLLIYEVSNR

Z27499|IGKV2D-2 EIVMTQTPLSLSITPGEQASMSCRSSQSLLHS-DGYTYLYWFLQKARPVSTLLICEVSNR

::****:*** .:* *: **:**:***** * * .** * *:. . ** *

Vedolizumab FSGVPDRFSGSGSGTDFTLKISRVEAEDVGVYYCLQGTHQPYTFGQGTKVEIKR

X12684|IGKV2-24 FSGVPDRFSGSGAGTDFTLKISRVEAEDVGVYYCMQATQFP-------------

X63403|IGKV2-30 DSGVPDRFSGSGSGTDFTLKISRVEAEDVGVYYCMQGTHWP-------------

FM164408|IGKV2- DSGVPDRFSGSGSGTDFTLKISRVEAEDVGVYYCMQGTHWP-------------

X63402|IGKV2D-3 DSGVPDRFSGSGSGTDFTLKISRVEAEDVGVYYCMQGTHWP-------------

X63397|IGKV2-28 ASGVPDRFSGSGSGTDFTLKISRVEAEDVGVYYCMQALQTP-------------

X12691|IGKV2D-2 ASGVPDRFSGSGSGTDFTLKISRVEAEDVGVYYCMQALQTP-------------

X63396|IGKV2-29 FSGVPDRFSGSGSGTDFTLKISRVEAEDVGVYY*MQGIHLP-------------

U41645|IGKV2-29 FSGVPDRFSGSGSGTDFTLKISRVEAEDVGVYYCMQGIHLP-------------

AJ783437|IGKV2- FSGVPDRFSGSGSGTDFTLKISRVEAEDVGVYYCMQGIHLP-------------

M31952|IGKV2D-2 FSGVPDRFSGSGSGTDFTLKISRVEAEDVGVYYCMQSIQLP-------------

U41644|IGKV2D-2 FSGVPDRFSGSGSGTDFTLKISRVEAEDVGVYYCMQSIQLP-------------

X59314|IGKV2-40 ASGVPDRFSGSGSGTDFTLKISRVEAEDVGVYYCMQRIEFP-------------

X59311|IGKV2D-4 ASGVPDRFSGSGSGTDFTLKISRVEAEDVGVYYCMQRIEFP-------------

AP001216|IGKV2D FSGVPDRFSGSGSGTDFTLKISRVEAEDFGVYYCMQDAQDP-------------

KM455565|IGKV2D FSGVPDRFSGSGSGTDFTLKISRVEAEDFGVYYCMQDAQDP-------------

Z27499|IGKV2D-2 FSGVPDRFSGSGSGTDFTLKISRVEAEDFGVYYCMQDAQDP-------------

***********:***************.**** :* . *

**Supplementary Data 5**. Multiple sequence alignment of available commercial and IMGT light chain family for Vκ2 framework determination. Framework are chosen based on similarity among its family members highlighted in green.

**Vκ3 Framework Determination**

Gemtuzumab QIVLTQSPAIMSASPGEKVTITCSASSSI-----SYMHWFQQKPGTSPKLWIYTTSNLAS

Matuzumab DIQMTQSPSSLSASVGDRVTITCSASSSV-----TYMYWYQQKPGKAPKLLIYDTSNLAS

Pembrolizumab EIVLTQSPATLSLSPGERATLSCRASKGVSTSGYSYLHWYQQKPGQAPRLLIYLASYLES

X01668|IGKV3-11 EIVLTQSPATLSLSPGERATLSCRASQSV-S---SYLAWYQQKPGQAPRLLIYDASNRAT

K02768|IGKV3-11 EIVLTQSPATLSLSPGERATLSCRASQSV-S---SYLAWYQQKPGQAPRLLIYDASNRAT

X17264|IGKV3D-1 EIVLTQSPATLSLSPGERATLSCRASQGV-S---SYLAWYQQKPGQAPRLLIYDASNRAT

KM455561|IGKV3D EIVLTQSPATLSLSPGERATLSCRASQSV-S---SYLAWYQQKPGQAPRLLIYDASNRAT

L19271|IGKV3D-1 EIVLTQSPATLSLSPGERATLSCRASQGV-S---SNLAWYQQKPGQAPRLLIYDASNRAT

X12686|IGKV3-20 EIVLTQSPGTLSLSPGERATLSCRASQSVSS---SYLAWYQQKPGQAPRLLIYGASSRAT

L37729|IGKV3-20 EIVLTQSPATLSLSPGERATLSCRASQSVSS---SYLAWYQQKPGQAPRLLIYGASSRAT

L19272|IGKV3D-2 EIVLTQSPATLSLSPGERATLSCRASQSVSS---SYLAWYQQKPGQAPRLLIYDASSRAT

X12687|IGKV3D-2 EIVLTQSPATLSLSPGERATLSCGASQSVSS---SYLAWYQQKPGLAPRLLIYDASSRAT

X72820|IGKV3D-7 EIVMTQSPATLSLSPGERATLSCRASQSVSS---SYLSWYQQKPGQAPRLLIYGASTRAT

M23090|IGKV3-15 EIVMTQSPATLSVSPGERATLSCRASQSV-S---SNLAWYQQKPGQAPRLLIYGASTRAT

X72815|IGKV3D-1 EIVMTQSPATLSVSPGERATLSCRASQSV-S---SNLAWYQQKPGQAPRLLIYGASTRAT

M23091|IGKV3D-1 EIVMMQSPATLSVSPGERATLSCRASQSV-S---SNLAWYQQKPGQAPRLLIYGASTRAT

KM455564|IGKV3D EIVMTQSPATLSVSPGERATLSCRASQSV-S---SNLAWYQQKPGQAPRLLIYGASIRAT

:* : ***. :* * *::.*::* **..: : : *:***** :*:* ** :* :

Gemtuzumab GVPARFSGSGSGTSYSLTISRMEAEDAATYYCHQRSTYPLTFGSGTKLELK-

Matuzumab GVPSRFSGSGSGTDYTFTISSLQPEDIATYYCQQWSSHIFTFGQGTKVEIKR

Pembrolizumab GVPARFSGSGSGTDFTLTISSLEPEDFAVYYCQHSRDLPLTFGGGTKVEIKR

X01668|IGKV3-11 GIPARFSGSGSGTDFTLTISSLEPEDFAVYYCQQRSNWP-------------

K02768|IGKV3-11 GIPARFSGSGSGRDFTLTISSLEPEDFAVYYCQQRSNWP-------------

X17264|IGKV3D-1 GIPARFSGSGPGTDFTLTISSLEPEDFAVYYCQQRSNWH-------------

KM455561|IGKV3D GIPARFSGSGPGTDFTLTISSLEPEDFAVYYCQQRSNWH-------------

L19271|IGKV3D-1 GIPARFSGSGPGTDFTLTISSLEPEDFAVYYCQQRSNWH-------------

X12686|IGKV3-20 GIPDRFSGSGSGTDFTLTISRLEPEDFAVYYCQQYGSSP-------------

L37729|IGKV3-20 GIPARFSGSGSGTDFTLTISRLEPEDFAVYYCQ-------------------

L19272|IGKV3D-2 GIPDRFSGSGSGTDFTLTISRLEPEDFAVYYCQQRSNWH-------------

X12687|IGKV3D-2 GIPDRFSGSGSGTDFTLTISRLEPEDFAVYYCQQYGSSP-------------

X72820|IGKV3D-7 GIPARFSGSGSGTDFTLTISSLQPEDFAVYYCQQDYNLP-------------

M23090|IGKV3-15 GIPARFSGSGSGTEFTLTISSLQSEDFAVYYCQQYNNWP-------------

X72815|IGKV3D-1 GIPARFSGSGSGTEFTLTISSLQSEDFAVYYCQQYNNWP-------------

M23091|IGKV3D-1 GIPARFSGSGSGTEFTLTISSLQSEDFAVYYCQQYNN*P-------------

KM455564|IGKV3D GIPARFSGSGSGTEFTLTISILQSEDFAVYYCQQYNNWP-------------

*:* ******.* .:::*** ::.** *.***:

**Supplementary Data 6**. Multiple sequence alignment of available commercial and IMGT light chain family for Vκ3 framework determination. Framework are chosen based on similarity among its family members highlighted in green.

**Vκ4 Framework Determination**

IGKV4-1*01| sapiens

DIVMTQSPDSLAVSLGERATINCKSSQSVLYSSNNKNYLAWYQQKPGQPPKLLIYWASTRESGVPDRFSGSGSGTDFTLTISSLQAEDVAVYYCQQYYSTP

**Supplementary Data 7**. Available IMGT light chain family for Vκ4 framework determination. Framework are chosen based on similarity among its family members highlighted in green.

**Vκ5 Framework Determination**

IGKV5-2*01| sapiens

ETTLTQSPAFMSATPGDKVNISCKASQDIDDDMNWYQQKPGEAAIFIIQEATTLVPGIPPRFSGSGYGTDFTLTINNIESEDAAYYFCLQHDNFP

**Supplementary Data 8**. Available IMGT light chain family for Vκ5 framework determination. Framework are chosen based on similarity among its family members highlighted in green.

**Vκ6 Framework Determination**

X63399|IGKV6-21 EIVLTQSPDFQSVTPKEKVTITCRASQSIGSSLHWYQQKPDQSPKLLIKYASQSFSGVPS

X12683|IGKV6D-2 EIVLTQSPDFQSVTPKEKVTITCRASQSIGSSLHWYQQKPDQSPKLLIKYASQSFSGVPS

KM455568|IGKV6- EIVLTQSPDFQSVTPKEKVTITCRASQSIGSSLHWYQQKPDQSPKLLIKYASQSISGVPS

KM455569|IGKV6D EIVLTQSPDFQSVTPKEKVTITCRASQSIGSSLHWYQQKPDQSPKLLIKYASQSISGVPS

******************************************************:*****

X63399|IGKV6-21 RFSGSGSGTDFTLTINSLEAEDAATYYCHQSSSLP

X12683|IGKV6D-2 RFSGSGSGTDFTLTINSLEAEDAATYYCHQSSSLP

KM455568|IGKV6- RFSGSGSGTDFTLTINSLEAEDAATYYCHQSSSLP

KM455569|IGKV6D RFSGSGSGTDFTLTINSLEAEDAAAYYCHQSSSLP

************************:**********

**Supplementary Data 9**. Available IMGT light chain family for Vκ6 framework determination. Framework are chosen based on similarity among its family members highlighted in green.

**VH CDR Determination**

Efalizumab EVQLVESGGGLVQPGGSLRLSCAASGYSFT-GHWMNWVRQAPGKGLEWVGIMIHPSDSET

Omalizumab EVQLVESGGGLVQPGGSLRLSCAVSGYSITSGYSWNWIRQAPGKGLEWVASI--TYDGST

Pertuzumab EVQLVESGGGLVQPGGSLRLSCAASGFTFT-DYTMDWVRQAPGKGLEWVADV-NPNSGGS

Trastuzumab EVQLVESGGGLVQPGGSLRLSCAASGFNIK-DTYIHWVRQAPGKGLEWVARI-YPTNGYT

Certolizumab EVQLVESGGGLVQPGGSLRLSCAASGYVFT-DYGMNWVRQAPGKGLEWMGWI-NTYIGEP

***********************.**: :. .*:**********:. : .

Efalizumab RYNQKFKDIRFTISVDKSKNTLYLQMNSLRAEDTAVYYCARIGIYFYGTTYFDYIWGQGT

Omalizumab NYADSVK-GRFTISRDDSKNTFYLQMNSLRAEDTAVYYCARGSHYF--GHWHFAVWGQGT

Pertuzumab IYNQRFK-GRFTLSVDRSKNTLYLQMNSLRAEDTAVYYCARNLG-P--SFY-FDYWGQGT

Trastuzumab RYADSVK-GRFTISADTSKNTAYLQMNSLRAEDTAVYYCSRWGG-D--GFYAMDYWGQGT

Certolizumab IYADSVK-GRFTFSLDTSKSTAYLQMNSLRAEDTAVYYCAR--G-Y--RSYAMDYWGQGT

* : .* ***:* * **.* *****************:* : *****

Efalizumab LVTVSS

Omalizumab LVTVSS

Pertuzumab LVTVSS

Trastuzumab LVTVSS

Certolizumab LVTVSS

******

**Supplementary Data 10**. Multiple sequence alignment of commercial heavy chain family for CDRs determination of Pertuzumab and Trastuzumab. Heavy chain of same family (VH3) with Pertuzumab and Trastuzumab were used for comparison. We determined that the most contrasting region belongs to CDR regions highlighted in red while Pertuzumab CDRs are highlighted in cyan and Trastuzumab CDRs are highlighted in yellow.

**VH1 Framework Determination**

Gemtuzumab QVQLQQSGAELAKPGASVKMSCKASGYTFTSYRMHWVKQRPGQGLEWIGYINPSTGYTEY

Daclizumab QVQLVQSGAEVKKPGSSVKVSCKASGYTFTSYRMHWVRQAPGQGLEWIGYINPSTGYTEY

M99642|IGHV1-24*01| QVQLVQSGAEVKKPGASVKVSCKVSGYTLTELSMHWVRQAPGKGLEWMGGFDPEDGETIY

KF698734|IGHV1-69-2*01| EVQLVQSGAEVKKPGATVKISCKVSGYTFTDYYMHWVQQAPGKGLEWMGLVDPEDGETIY

Pembrolizumab QVQLVQSGVEVKKPGASVKVSCKASGYTFTNYYMYWVRQAPGQGLEWMGGINPSNGGTNF

Matuzumab QVQLVQSGAEVKKPGASVKVSCKASGYTFTSHWMHWVRQAPGQGLEWIGEFNPSNGRTNY

M29809|IGHV1-58*01| QMQLVQSGPEVKKPGTSVKVSCKASGFTFTSSAVQWVRQARGQRLEWIGWIVVGSGNTNY

AB019438|IGHV1-58*02| QMQLVQSGPEVKKPGTSVKVSCKASGFTFTSSAMQWVRQARGQRLEWIGWIVVGSGNTNY

Natalizumab QVQLVQSGAEVKKPGASVKVSCKASGFNIKDTYIHWVRQAPGQRLEWMGRIDPANGYTKY

X92209|IGHV1-45*01| QMQLVQSGAEVKKTGSSVKVSCKASGYTFTYRYLHWVRQAPGQALEWMGWITPFNGNTNY

AB019438|IGHV1-45*02| QMQLVQSGAEVKKTGSSVKVSCKASGYTFTYRYLHWVRQAPGQALEWMGWITPFNGNTNY

Z14300|IGHV1-69*10| QVQLVQSGAEVKKPGSSVKVSCKASGGTFSSYAISWVRQAPGQGLEWMGGIIPILGIANY

Z14309|IGHV1-69*08| QVQLVQSGAEVKKPGSSVKVSCKASGGTFSSYTISWVRQAPGQGLEWMGRIIPILGTANY

Z27506|IGHV1-69*02| QVQLVQSGAEVKKPGSSVKVSCKASGGTFSSYTISWVRQAPGQGLEWMGRIIPILGIANY

M83132|IGHV1-69*04| QVQLVQSGAEVKKPGSSVKVSCKASGGTFSSYAISWVRQAPGQGLEWMGRIIPILGIANY

Z14307|IGHV1-69*09| QVQLVQSGAEVKKPGSSVKVSCKASGGTFSSYAISWVRQAPGQGLEWMGRIIPILGIANY

Z14296|IGHV1-69*11| QVQLVQSGAEVKKPGSSVKVSCKASGGTFSSYAISWVRQAPGQGLEWMGRIIPILGTANY

X92340|IGHV1-69*03| QVQLVQSGAEVKKPGSSVKVSCKASGGTFSSYAISWVRQAPGQGLEWMGGIIPIFGTANY

L22583|IGHV1-69*06| QVQLVQSGAEVKKPGSSVKVSCKASGGTFSSYAISWVRQAPGQGLEWMGGIIPIFGTANY

KC713948|IGHV1-69*14| QVQLVQSGAEVKKPGSSVKVSCKASGGTFSSYAISWVRQAPGQGLEWMGGIIPIFGTANY

L22582|IGHV1-69*01| QVQLVQSGAEVKKPGSSVKVSCKASGGTFSSYAISWVRQAPGQGLEWMGGIIPIFGTANY

Z14301|IGHV1-69*12| QVQLVQSGAEVKKPGSSVKVSCKASGGTFSSYAISWVRQAPGQGLEWMGGIIPIFGTANY

Z14214|IGHV1-69*13| QVQLVQSGAEVKKPGSSVKVSCKASGGTFSSYAISWVRQAPGQGLEWMGGIIPIFGTANY

KC713934|IGHV1-69D*01| QVQLVQSGAEVKKPGSSVKVSCKASGGTFSSYAISWVRQAPGQGLEWMGGIIPIFGTANY

X67905|IGHV1-69*05| QVQLVQSGAEVKKPGSSVKVSCKASGGTFSSYAISWVRQAPGQGLEWMGGIIPIFGTANY

Vedolizumab QVQLVQSGAEVKKPGASVKVSCKGSGYTFTSYWMHWVRQAPGQRLEWIGEIDPSESNTNY

HM855463|IGHV1-18*03| QVQLVQSGAEVKKPGASVKVSCKASGYTFTSYGISWVRQAPGQGLEWMGWISAYNGNTNY

M99641|IGHV1-18*01| QVQLVQSGAEVKKPGASVKVSCKASGYTFTSYGISWVRQAPGQGLEWMGWISAYNGNTNY

X60503|IGHV1-18*02| QVQLVQSGAEVKKPGASVKVSCKASGYTFTSYGISWVRQAPGQGLEWMGWISAYNGNTNY

KC713938|IGHV1-18*04| QVQLVQSGAEVKKPGASVKVSCKASGYTFTSYGISWVRQAPGQGLEWMGWISAYNGNTNY

X62109|IGHV1-3*01| QVQLVQSGAEVKKPGASVKVSCKASGYTFTSYAMHWVRQAPGQRLEWMGWINAGNGNTKY

X62107|IGHV1-3*02| QVQLVQSGAEVKKPGASVKVSCKASGYTFTSYAMHWVRQAPGQRLEWMGWSNAGNGNTKY

Pexelizumab QVQLVQSGAEVKKPGASVKVSCKASGYIFSNYWIQWVRQAPGQGLEWMGEILPGSGSTEY

X92343|IGHV1-46*01| QVQLVQSGAEVKKPGASVKVSCKASGYTFTSYYMHWVRQAPGQGLEWMGIINPSGGSTSY

L06612|IGHV1-46*03| QVQLVQSGAEVKKPGASVKVSCKASGYTFTSYYMHWVRQAPGQGLEWMGIINPSGGSTSY

J00240|IGHV1-46*02| QVQLVQSGAEVKKPGASVKVSCKASGYTFNSYYMHWVRQAPGQGLEWMGIINPSGGSTSY

M99637|IGHV1-8*01| QVQLVQSGAEVKKPGASVKVSCKASGYTFTSYDINWVRQATGQGLEWMGWMNPNSGNTGY

HM855457|IGHV1-8*02| QVQLVQSGAEVKKPGASVKVSCKASGYTFTSYDINWVRQATGQGLEWMGWMNPNSGNTGY

X07448|IGHV1-2*01| QVQLVQSGAEVKKPGASVKVSCKASGYTFTGYYMHWVRQAPGQGLEWMGRINPNSGGTNY

HM855674|IGHV1-2*05| QVQLVQSGAEVKKPGASVKVSCKASGYTFTGYYMHWVRQAPGQGLEWMGRINPNSGGTNY

X92208|IGHV1-2*03| QVQLVQSGAEVKKLGASVKVSCKASGYTFTGYYMHWVXQAPGQGLEWMGWINPNSGGTNY

X62106|IGHV1-2*02| QVQLVQSGAEVKKPGASVKVSCKASGYTFTGYYMHWVRQAPGQGLEWMGWINPNSGGTNY

KF698733|IGHV1-2*04| QVQLVQSGAEVKKPGASVKVSCKASGYTFTGYYMHWVRQAPGQGLEWMGWINPNSGGTNY

::** *** *: * *::**:*** ** :. : ** * *: ***:* . : :

Gemtuzumab NQKFKDKATLTADKSSSTAYMQLSSLTFEDSAVYYCARGG-------GVFDYWGQGTTLT

Daclizumab NQKFKDKATITADESTNTAYMELSSLRSEDTAVYYCARGG-------GVFDYWGQGTTLT

M99642|IGHV1-24*01| AQKFQGRVTMTEDTSTDTAYMELSSLRSEDTAVYYCAT----------------------

KF698734|IGHV1-69-2*01| AEKFQGRVTITADTSTDTAYMELSSLRSEDTAVYYCAT----------------------

Pembrolizumab NEKFKNRVTLTTDSSTTTAYMELKSLQFDDTAVYYCARRDYRF---DMGFDYWGQGTTVT

Matuzumab NEKFKSKATMTVDTSTNTAYMELSSLRSEDTAVYYCASRDYD--YDGRYFDYWGQGTLVT

M29809|IGHV1-58*01| AQKFQERVTITRDMSTSTAYMELSSLRSEDTAVYYCAA----------------------

AB019438|IGHV1-58*02| AQKFQERVTITRDMSTSTAYMELSSLRSEDTAVYYCAA----------------------

Natalizumab DPKFQGRVTITADTSASTAYMELSSLRSEDEAVYYCAREGYYGNYGVYAMDYWGQGTLVT

X92209|IGHV1-45*01| AQKFQDRVTITRDRSMSTAYMELSSLRSEDTAMYYCAR----------------------

AB019438|IGHV1-45*02| AQKFQDRVTITRDRSMSTAYMELSSLRSEDTAMYYCAR----------------------

Z14300|IGHV1-69*10| AQKFQGRVTITADKSTSTAYMELSSLRSEDTAVYYCAR----------------------

Z14309|IGHV1-69*08| AQKFQGRVTITADKSTSTAYMELSSLRSEDTAVYYCAR----------------------

Z27506|IGHV1-69*02| AQKFQGRVTITADKSTSTAYMELSSLRSEDTAVYYCAR----------------------

M83132|IGHV1-69*04| AQKFQGRVTITADKSTSTAYMELSSLRSEDTAVYYCAR----------------------

Z14307|IGHV1-69*09| AQKFQGRVTITADKSTSTAYMELSSLRSEDTAVYYCAR----------------------

Z14296|IGHV1-69*11| AQKFQGRVTITADESTSTAYMELSSLRSEDTAVYYCAR----------------------

X92340|IGHV1-69*03| AQKFQGRVTITADESTSTAYMELSSLRSDDT-----------------------------

L22583|IGHV1-69*06| AQKFQGRVTITADKSTSTAYMELSSLRSEDTAVYYCAR----------------------

KC713948|IGHV1-69*14| AQKFQGRVTITADKSTSTAYMELSSLRSEDTAVYYCAR----------------------

L22582|IGHV1-69*01| AQKFQGRVTITADESTSTAYMELSSLRSEDTAVYYCAR----------------------

Z14301|IGHV1-69*12| AQKFQGRVTITADESTSTAYMELSSLRSEDTAVYYCAR----------------------

Z14214|IGHV1-69*13| AQKFQGRVTITADESTSTAYMELSSLRSEDTAVYYCAR----------------------

KC713934|IGHV1-69D*01| AQKFQGRVTITADESTSTAYMELSSLRSEDTAVYYCAR----------------------

X67905|IGHV1-69*05| AQKFQGRVTITTDESTSTAYMELSSLRSEDTAVYYCAR----------------------

Vedolizumab NQKFKGRVTLTVDISASTAYMELSSLRSEDTAVYYCARGGYDGW--DYAIDYWGQGTLVT

HM855463|IGHV1-18*03| AQKLQGRVTMTTDTSTSTAYMELRSLRSDDMAVYYCAR----------------------

M99641|IGHV1-18*01| AQKLQGRVTMTTDTSTSTAYMELRSLRSDDTAVYYCAR----------------------

X60503|IGHV1-18*02| AQKLQGRVTMTTDTSTSTAYMELRSLRSDDTA----------------------------

KC713938|IGHV1-18*04| AQKLQGRVTMTTDTSTSTAYMELRSLRSDDTAVYYCAR----------------------

X62109|IGHV1-3*01| SQKFQGRVTITRDTSASTAYMELSSLRSEDTAVYYCAR----------------------

X62107|IGHV1-3*02| SQEFQGRVTITRDTSASTAYMELSSLRSEDMAVYYCAR----------------------

Pexelizumab AQKFQGRVTMTADTSTSTAYMELSSLRSEDTAVYYCARYFFGSS--PNWYFVWGQGT---

X92343|IGHV1-46*01| AQKFQGRVTMTRDTSTSTVYMELSSLRSEDTAVYYCAR----------------------

L06612|IGHV1-46*03| AQKFQGRVTMTRDTSTSTVYMELSSLRSEDTAVYYCAR----------------------

J00240|IGHV1-46*02| AQKFQGRVTMTRDTSTSTVYMELSSLRSEDTAVYYCAR----------------------

M99637|IGHV1-8*01| AQKFQGRVTMTRNTSISTAYMELSSLRSEDTAVYYCAR----------------------

HM855457|IGHV1-8*02| AQKFQGRVTMTRNTSISTAYMELSSLRSEDTAVYYCAR----------------------

X07448|IGHV1-2*01| AQKFQGRVTSTRDTSISTAYMELSRLRSDDTVVYYCAR----------------------

HM855674|IGHV1-2*05| AQKFQGRVTMTRDTSISTAYMELSRLRSDDTVVYYCAR----------------------

X92208|IGHV1-2*03| AQKFQGRVTMTRDTSISTAYMELSRLRSDDTAVYYCAR----------------------

X62106|IGHV1-2*02| AQKFQGRVTMTRDTSISTAYMELSRLRSDDTAVYYCAR----------------------

KF698733|IGHV1-2*04| AQKFQGWVTMTRDTSISTAYMELSRLRSDDTAVYYCAR----------------------

::: .* * : * *.**:* * :*

**Supplementary Data 11**. Multiple sequence alignment of available commercial and IMGT heavy chain family for VH1 framework determination. Framework are chosen based on similarity among its family members highlighted in green.

**VH2 Framework Determination**

M99648|IGHV2-26*01| QVTLKESGPVLVKPTETLTLTCTVSGFSLSNARMGVSWIRQPPGKALEWLAHIFSNDEKS

Palivizumab QVTLRESGPALVKPTQTLTLTCTFSGFSLSTSGMSVGWIRQPPGKALEWLADIWWDDKKD

L21971|IGHV2-5*08| QVTLKESGPALVKPTQTLTLTCTFSGFSLSTSGMRVSWIRQPPGKALEWLALIYWDDDKR

L21964|IGHV2-5*05| QITLKESGPTLVKPTQTLTLTCTFSGFSLSTSGVGVGWIRQPPGKALEWLALIYWDDDKR

L21966|IGHV2-5*06| QITLKESGPTLVKPTQTLTLTCTFSGFSLSTSGVGVGWIRQPPGKALEWLALIYWDDDKR

L21972|IGHV2-5*09| QVTLKESGPTLVKPTQTLTLTCTFSGFSLSTSGVGVGWIRQPPGKALEWLALIYWDDDKR

L21963|IGHV2-5*04| QITLKESGPTLVKPTQTLTLTCTFSGFSLSTSGVGVGWIRQPPGKALEWLALIYWNDDKR

X62111|IGHV2-5*01| QITLKESGPTLVKPTQTLTLTCTFSGFSLSTSGVGVGWIRQPPGKALEWLALIYWNDDKR

KF698731|IGHV2-5*02| QITLKESGPTLVKPTQTLTLTCTFSGFSLSTSGVGVGWIRQPPGKALEWLALIYWDDDKR

X93619|IGHV2-5*03| ----------LVKPTQTLTLTCTFSGFSLSTSGVGVGWIRQPPGKALEWLALIYWDDDKR

L21962|IGHV2-70*09| QITLKESGPTLVKPTQTLTLTRTFSGFSLSTSGMCVSWIRQPPGKALEWLALIDWDDDKY

L21970|IGHV2-70*12| QITLKESGPTLVKPTQTLTLTCTFSGFSLSTSGMCVSWIRQPPGKALEWLALIDWDDDKY

L21965|IGHV2-70*10| QVTLKESGPALVKPTQTLTLTCTFSGFSLSTSGMRVSWIRQPPGKALEWIARIDWDDDKY

X92238|IGHV2-70*03| QVTLKESGPALVKPTQTLTLTCTFSGFSLSTSGMRVSWIRQPPGKALEWLARIDWDDDKF

X92239|IGHV2-70*06| QVTLKESGPALVKPTQTLTLTCTFSGFSLSTSGMRVSWIRQPPGKALEWLARIDWDDDKF

Z12330|IGHV2-70*04| QVTLKESGPALVKPTQTLTLTCTFSGFSLSTSGMRVSWIRQPPGKALEWLARIDWDDDKF

KC713935|IGHV2-70D*04| QVTLKESGPALVKPTQTLTLTCTFSGFSLSTSGMRVSWIRQPPGKALEWLARIDWDDDKF

KC713949|IGHV2-70D*14| QVTLKESGPALVKPTQTLTLTCTFSGFSLSTSGMRVSWIRQPPGKALEWLARIDWDDDKF

Z27502|IGHV2-70*05| ---------ALVKPTQTLTLTCTFSGFSLSTSGMRASWIRQPPGKALEWLARIDWDDDKF

X92245|IGHV2-70*08| QVTLRESGPALVKPTQTLTLTCAFSGFSLSTSGMCVSWIRQPPGKALEWLARIDWDDDKY

L21967|IGHV2-70*11| RVTLRESGPALVKPTQTLTLTCTFSGFSLSTSGMCVSWIRQPPGKALEWLARIDWDDDKY

L21969|IGHV2-70*01| QVTLRESGPALVKPTQTLTLTCTFSGFSLSTSGMCVSWIRQPPGKALEWLALIDWDDDKY

AB019437|IGHV2-70*13| QVTLRESGPALVKPTQTLTLTCTFSGFSLSTSGMCVSWIRQPPGKALEWLALIDWDDDKY

X92241|IGHV2-70*02| QVTLRESGPALVKPTQTLTLTCTFSGFSLSTSGMCVSWIRQPPGKALEWLALIDWDDDKY

X92243|IGHV2-70*07| QVTLRESGPALVKPTQTLTLTCTFSGFSLSTSGMCVSWIRQPPGKALEWLALIDWDDDKY

*****:***** :.******.: : ..************:* * :*.*

M99648|IGHV2-26*01| YSTSLKSRLTISKDTSKSQVVLTMTNMDPVDTATYYCARI--------------------

Palivizumab YNPSLKSRLTISKDTSKNQVVLKVTNMDPADTATYYCARSMITNWYFDVWGAGTTVTVSS

L21971|IGHV2-5*08| YSPSLKSRLTITKDTSKNQVVLTMTNMDPVDTATYYCAHR--------------------

L21964|IGHV2-5*05| YGPSLKSRLTITKDTSKNQVVLTMTNMDPVDTATYYCAHR--------------------

L21966|IGHV2-5*06| YGPSLKSRLTITKDTSKNQVVLTMTNMDPVDTATYYCAHR--------------------

L21972|IGHV2-5*09| YGPSLKSRLTITKDTSKNQVVLTMTNMDPVDTATYYCAHR--------------------

L21963|IGHV2-5*04| YSPSLKSRLTITKDTSKNQVVLTMTNMDPVDTGTYYCV----------------------

X62111|IGHV2-5*01| YSPSLKSRLTITKDTSKNQVVLTMTNMDPVDTATYYCAHR--------------------

KF698731|IGHV2-5*02| YSPSLKSRLTITKDTSKNQVVLTMTNMDPVDTATYYCAHR--------------------

X93619|IGHV2-5*03| YSPSLKSRLTITKDTSKNQ-----------------------------------------

L21962|IGHV2-70*09| YSTSLNTRLTISKDTSKNQVVLTMTNMDPVDTGTYYCVR---------------------

L21970|IGHV2-70*12| YSTSLKTRLTISKDTSKNQVVLTMTNMDPVDTATYYCAHR--------------------

L21965|IGHV2-70*10| YSTSLKTRLTISKDTSKNQVVLTMTNMDPVDTATYYCARI--------------------

X92238|IGHV2-70*03| YSTSLKTRLTISKDTSKNQVVLTMTNMDPVDTAVYY------------------------

X92239|IGHV2-70*06| YSTSLKTRLTISKDTSKNQVVLTMTNMDPVDTAVYY------------------------

Z12330|IGHV2-70*04| YSTSLKTRLTISKDTSKNQVVLTMTNMDPVDTATYY------------------------

KC713935|IGHV2-70D*04| YSTSLKTRLTISKDTSKNQVVLTMTNMDPVDTATYYCARI--------------------

KC713949|IGHV2-70D*14| YSTSLKTRLTISKDTSKNQVVLTMTNMDPVDTATYYCARI--------------------

Z27502|IGHV2-70*05| YSTSLKTRLTISKDTSKNQVVLTMTNM---------------------------------

X92245|IGHV2-70*08| YSTSLKTRLTISKDTSKNQVVLTMTNMDPVDTAVYY------------------------

L21967|IGHV2-70*11| YSTSLKTRLTISKDTSKNQVVLTMTNMDPVDTATYYCARI--------------------

L21969|IGHV2-70*01| YSTSLKTRLTISKDTSKNQVVLTMTNMDPVDTATYYCARI--------------------

AB019437|IGHV2-70*13| YSTSLKTRLTISKDTSKNQVVLTMTNMDPVDTATYYCARI--------------------

X92241|IGHV2-70*02| YSTSLKTRLTISKDTSKNQVVLTMTNMDPVDTAVYY------------------------

X92243|IGHV2-70*07| YSTSLKTRLTISKDTSKNQVVLTMTNMDPVDTAVYY------------------------

* **::****:*****.*

**Supplementary Data 12**. Multiple sequence alignment of available commercial and IMGT heavy chain family for VH2 framework determination. Framework are chosen based on similarity among its family members highlighted in green.

**VH4 Framework Determination**

Cetuximab QVQLKQSGPGLVQPSQSLSITCTVSGFSLT--NYGVHWVRQSPGKGLEWLGVIWSGG---

Alemtuzumab QVQLQESGPGLVRPSQTLSLTCTVSGFTFT--DFYMNWVRQPPGRGLEWIGFIRDKAKGY

Z14241|IGHV4-34*09| QVQLQESGPGLVKPSQTLSLTCAVYGGSFS--GYYWSWIRQPPGKGLEWIGEINHSG---

Z14242|IGHV4-34*10| QVQLQESGPGLVKPSETLSLTCAVYGGSFS--GYYWSWIRQPPGKGLEWIGEINHSG---

X05716|IGHV4-34*11| QVQLQQWGAGLLKPSETLSLTCAVYGGSVS--GYYWSWIRQPPGKGLEWIGYIYYSG---

X92237|IGHV4-34*05| QVQLQQWGAGLLKPSETLSLTCAVYGGSFS--GYYWCWIRQPLGKGLEWIGEINHSG---

X92236|IGHV4-34*04| QVQLQQWGAGLLKPSETLSLTCAVYGGSFS--GYYWSWIRQPPGKGLEWIGEINHSG---

X92256|IGHV4-34*06| QVQLQQWGAGLLKPSETLSLTCAVYGGSFS--GYYWSWIRQPPGKGLEWIGEINHSG---

M95113|IGHV4-34*08| QVQLQQWGAGLLKPSETLSLTCAVYGGTFS--GYYWSWIRQPPGKGLEWIGEINHSG---

X56591|IGHV4-34*12| QVQLQQWGAGLLKPSETLSLTCAVYGGSFS--GYYWSWIRQPPGKGLEWIGEIIHSG---

AB019439|IGHV4-34*01| QVQLQQWGAGLLKPSETLSLTCAVYGGSFS--GYYWSWIRQPPGKGLEWIGEINHSG---

M99684|IGHV4-34*02| QVQLQQWGAGLLKPSETLSLTCAVYGGSFS--GYYWSWIRQPPGKGLEWIGEINHSG---

X92255|IGHV4-34*03| QVQLQQWGAGLLKPSETLSLTCAVYGGSFS--GYYWSWIRQPPGKGLEWIGEINHSG---

X92258|IGHV4-34*07| QVQLQQWGAGLLKPSETLSLTCAVYGGSFS--GYYWSWIRQPPGKGLEWIGEINHSG---

X92254|IGHV4-4*05| QVQLQELGPGLVKPPGTLSLTCAVSGGSISS-SNWWSWVRQPPGKGLEWIGEIYHSG---

X92253|IGHV4-4*04| QVQLQESGPGLVKPPGTLSLTCAISGGSISS-SNWWSWVRQPPGKGLEWIGEIYHSG---

X92232|IGHV4-4*02| QVQLQESGPGLVKPSGTLSLTCAVSGGSISS-SNWWSWVRQPPGKGLEWIGEIYHSG---

X05713|IGHV4-4*01| QVQLQESGPGLVKPPGTLSLTCAVSGGSISS-SNWWSWVRQPPGKGLEWIGEIYHSG---

X92252|IGHV4-4*03| QVQLQESGPGLVKPPGTLSLTCAVSGGSISS-SNWWSWVRQPPGKGLEWIGEIYHSG---

X56356|IGHV4-61*05| QLQLQESGPGLVKPSETLSLTCTVSGGSISSSSYYWGWIRQPPGKGLEWIGYIYYSG---

Z14236|IGHV4-39*06| RLQLQESGPGLVKPSETLSLTCTVSGGSISSSSYYWGWIRQPPGKGLEWIGSIYYSG---

M95116|IGHV4-39*05| QLQLQESGPGLVKPSETPSLTCTVSGGSISSSSYYWGWIRQPPGKGLEWIGSIYYSG---

X05715|IGHV4-39*02| QLQLQESGPGLVKPSETLSLTCTVSGGSISSSSYYWGWIRQPPGKGLEWIGSIYYSG---

AB019439|IGHV4-39*01| QLQLQESGPGLVKPSETLSLTCTVSGGSISSSSYYWGWIRQPPGKGLEWIGSIYYSG---

X92259|IGHV4-39*03| QLQLQESGPGLVKPSETLSLTCTVSGGSISSSSYYWGWIRQPPGKGLEWIGSIYYSG---

AM940222|IGHV4-39*07| QLQLQESGPGLVKPSETLSLTCTVSGGSISSSSYYWGWIRQPPGKGLEWIGSIYYSG---

X92250|IGHV4-61*04| QVQLQESGPGLVKPSETLSLTCTVSGGSVSSGSYYWSWIRQPPGKGLEWIGYIYYSG---

AB019437|IGHV4-61*08| QVQLQESGPGLVKPSETLSLTCTVSGGSVSSGGYYWSWIRQPPGKGLEWIGYIYYSG---

M29811|IGHV4-61*01| QVQLQESGPGLVKPSETLSLTCTVSGGSVSSGSYYWSWIRQPPGKGLEWIGYIYYSG---

X92230|IGHV4-61*03| QVQLQESGPGLVKPSETLSLTCTVSGGSVSSGSYYWSWIRQPPGKGLEWIGYIYYSG---

KC713946|IGHV4-30-4*07| QVQLQESGPGLVKPSQTLSLTCAVSGGSISSGGYSWSWIRQPPGKGLEWIGYIYYSG---

X92229|IGHV4-30-2*03| QLQLQESGSGLVKPSQTLSLTCAVSGGSISSGGYSWSWIRQPPGKGLEWIGSIYYSG---

KC713944|IGHV4-30-2*06| QLQLQESGSGLVKPSQTLSLTCAVSGGSISSGGYSWSWIRQSPGKGLEWIGYIYHSG---

HM855593|IGHV4-30-2*05| QLQLQESGSGLVKPSQTLSLTCAVSGGSISSGGYSWSWIRQPPGKGLEWIGYIYHSG---

L10089|IGHV4-30-2*01| QLQLQESGSGLVKPSQTLSLTCAVSGGSISSGGYSWSWIRQPPGKGLEWIGYIYHSG---

M95122|IGHV4-30-2*02| QLQLQESGSGLVKPSQTLSLTCAVSGGSISSGGYSWSWIRQPPGKGLEWIGYIYHSG---

L10097|IGHV4-61*02| QVQLQESGPGLVKPSQTLSLTCTVSGGSISSGSYYWSWIRQPAGKGLEWIGRIYTSG---

Z14235|IGHV4-31*10| QVQLQESGPGLLKPSQTLSLTCTVSGGSISSGGYYWSWIRQHPGKGLEWIGCIYYSG---

M95121|IGHV4-31*05| QVQLQESGPGLVKPSQTLSLTCTVSGGSISSGGYYWSWIRQHPGKGLEWIGYIYYSG---

X92273|IGHV4-31*09| QVQLQESGPGLVKPSQTLSLTCTVSGGSISSGGYYWSWIRQHPGKGLEWIGYIYYSG---

X92270|IGHV4-31*06| QVQLQESGPGLVKPSQTLSLTCTVSGGSISSGSYYWSWIRQHPGKGLEWIGYIYYSG---

M95120|IGHV4-31*04| QVRLQESGPGLVKPSQTLSLTCTVSGGSISSGGYYWSWIRQHPGKGLEWIGYIYYSG---

L10098|IGHV4-31*01| QVQLQESGPGLVKPSQTLSLTCTVSGGSISSGGYYWSWIRQHPGKGLEWIGYIYYSG---

M99683|IGHV4-31*02| QVQLQESGPGLVKPSQTLSLTCTVSGGSISSGGYYWSWIRQHPGKGLEWIGYIYYSG---

Z14237|IGHV4-31*03| QVQLQESGPGLVKPSQTLSLTCTVSGGSISSGGYYWSWIRQHPGKGLEWIGYIYYSG---

X92271|IGHV4-31*07| QVQLQESGPGLVKPSQTLSLTCTVSGGSISSGGYYWSWIRQHPGKGLEWIGYIYYSG---

X92272|IGHV4-31*08| QVQLQESGPGLVKPSQTLSLTCTVSGGSISSGGYYWSWIRQHPGKGLEWIGYIYYSG---

X92275|IGHV4-30-4*04| QVQLQDSGPGLVKPSQTLSLTCTVSGGSISSGDYYWSWIRQPPGKGLEWIGYFYYSG---

Z14239|IGHV4-30-4*02| QVQLQESGPGLVKPSDTLSLTCTVSGGSISSGDYYWSWIRQPPGKGLEWIGYIYYSG---

Z14238|IGHV4-30-4*01| QVQLQESGPGLVKPSQTLSLTCTVSGGSISSGDYYWSWIRQPPGKGLEWIGYIYYSG---

X92274|IGHV4-30-4*03| QVQLQESGPGLVKPSQTLSLTCTVSGGSISSGDYYWSWIRQPPGKGLEWIGYIYYSG---

HM855782|IGHV4-28*06| QVQLQESGPGLVKPSDTLSLTCAVSGYSISS-SNWWGWIRQPPGKGLEWIGYIYYSG---

X05714|IGHV4-28*01| QVQLQESGPGLVKPSDTLSLTCAVSGYSISS-SNWWGWIRQPPGKGLEWIGYIYYSG---

X92233|IGHV4-28*03| QVQLQESGPGLVKPSDTLSLTCAVSGYSISS-SNWWGWIRQPPGKGLEWIGYIYYSG---

KC713936|IGHV4-28*07| QVQLQESGPGLVKPSDTLSLTCAVSGYSISS-SNWWGWIRQPPGKGLEWIGYIYYSG---

X56358|IGHV4-28*04| QVQLQESGPGLVKPSDTLSLTCAVSGYSISS-SNWWGWIRQPPGKGLEWIGYIYYSG---

M83133|IGHV4-28*02| QVQLQESGPGLVKPSQTLSLTCAVSGYSISS-SNWWGWIRQPPGKGLEWIGYIYYSG---

HM855339|IGHV4-28*05| QVQLQESGPGLVKPSDTLSLTCAVSGYSISS-SNWWGWIRQPPGKGLEWIGYIYYSG---

Z12367|IGHV4-38-2*01| QVQLQESGPGLVKPSETLSLTCAVSGYSISS-GYYWGWIRQPPGKGLEWIGSIYHSG---

AC233755|IGHV4-38-2*02| QVQLQESGPGLVKPSETLSLTCTVSGYSISS-GYYWGWIRQPPGKGLEWIGSIYHSG---

Z14243|IGHV4-59*10| QVQLQQWGAGLLKPSETLSLTCAVYGGSIS--SYYWSWIRQPAGKGLEWIGRIYTSG---

M95119|IGHV4-59*06| QVQLQESGPGLVKPSETLSLTCTVTGGSIS--SYYWSWIRQPAGKGLEWIGYIYYSG---

X62112|IGHV4-4*07| QVQLQESGPGLVKPSETLSLTCTVSGGSIS--SYYWSWIRQPAGKGLEWIGRIYTSG---

M95118|IGHV4-59*05| QVQLQESGPGLVKPSETLSLTCTVSGGSIS--SYYWSWIRQPPGKGLEWIGRIYYSG---

M95117|IGHV4-59*04| QVQLQESGPGLVKPSETLSLTCTVSGGSIS--SYYWSWIRQPPGKGLEWIGYIYYSG---

X56360|IGHV4-59*07| QVQLQESGPGLVKPSDTLSLTCTVSGGSIS--SYYWSWIRQPPGKGLEWIGYIYYSG---

M29812|IGHV4-59*02| QVQLQESGPGLVKPSETLSLTCTVSGGSVS--SYYWSWIRQPPGKGLEWIGYIYYSG---

KC713942|IGHV4-4*08| QVQLQESGPGLVKPSETLSLTCTVSGGSIS--SYYWSWIRQPPGKGLEWIGYIYTSG---

AB019438|IGHV4-59*01| QVQLQESGPGLVKPSETLSLTCTVSGGSIS--SYYWSWIRQPPGKGLEWIGYIYYSG---

M95114|IGHV4-59*03| QVQLQESGPGLVKPSETLSLTCTVSGGSIS--SYYWSWIRQPPGKGLEWIGYIYYSG---

HM855471|IGHV4-59*08| QVQLQESGPGLVKPSETLSLTCTVSGGSIS--SYYWSWIRQPPGKGLEWIGYIYYSG---

:::*:: * **::* : *:**:: * :.: *:** *:****:* : .

Cetuximab NTDYNTPFTSRLSINKDNSKSQVFFKMNSLQSNDTAIYYCARALTYYDYEFAYWGQGTLV

Alemtuzumab TTEYNPSVKGRVTMLVDTSKNQFSLRLSSVTAADTAVYYCAREGH-TAAPFDYWGQGSLV

Z14241|IGHV4-34*09| STNYNPSLKSRVTISVDTSKNQFSLKLSSVTAADTAVYYCAR------------------

Z14242|IGHV4-34*10| STNYNPSLKSRITMSVDTSKNQFYLKLSSVTAADTAVYYCAR------------------

X05716|IGHV4-34*11| STNNNPSLKSRATISVDTSKNQFSLNLSSVTAADTAVYCCAR------------------

X92237|IGHV4-34*05| STNNNPSLKSRATISVDTSKNQFSLKLSSVTAADTAVYYCAR------------------

X92236|IGHV4-34*04| STNNNPSLKSRATISVDTSKNQFSLKLSSVTAADTAVYYCAR------------------

X92256|IGHV4-34*06| STNYNPSLKSRVTISVDTSKNQFSLKLGSVTAADTAVYY---------------------

M95113|IGHV4-34*08| STNYNPSLKSRVTISVDTSKNQFSLKLSSVTAADTAVYYCA-------------------

X56591|IGHV4-34*12| STNYNPSLKSRVTISVDTSKNQFSLKLSSVTAADTAVYYCAR------------------

AB019439|IGHV4-34*01| STNYNPSLKSRVTISVDTSKNQFSLKLSSVTAADTAVYYCAR------------------

M99684|IGHV4-34*02| STNYNPSLKSRVTISVDTSKNQFSLKLSSVTAADTAVYYCAR------------------

X92255|IGHV4-34*03| STNYNPSLKSRVTISVDTSKNQFSLKLSSVTAADTAVYY---------------------

X92258|IGHV4-34*07| STNYNPSLKSRVTISVDTSKNQFSLKLSSVTAADTAVYY---------------------

X92254|IGHV4-4*05| STNYNPSLKSRVTISVDKSKNQFSLKLSSVTAADTAVYY---------------------

X92253|IGHV4-4*04| STNYNPSLKSRVTISVDKSKNQFSLKLSSVTAADTAVYY---------------------

X92232|IGHV4-4*02| STNYNPSLKSRVTISVDKSKNQFSLKLSSVTAADTAVYYCAR------------------

X05713|IGHV4-4*01| STNYNPSLKSRVTISVDKSKNQFSLKLSSVTAADTAVYCCAR------------------

X92252|IGHV4-4*03| STNYNPSLKSRVTISVDKSKNQFSLKLSSVTAADTAVYY---------------------

X56356|IGHV4-61*05| STNYNPSLKSRVTISVDKSKNQFSLKLSSVTAADTAVYYCAR------------------

Z14236|IGHV4-39*06| STYYNPSLKSRVTISVDTSKNQFPLKLSSVTAADTAVYYCAR------------------

M95116|IGHV4-39*05| STYYNPSLKSRVTISVDTSKNQFSLKLSSVTAADTAVYYCA-------------------

X05715|IGHV4-39*02| STYYNPSLKSRVTISVDTSKNHFSLKLSSVTAADTAVYYCAR------------------

AB019439|IGHV4-39*01| STYYNPSLKSRVTISVDTSKNQFSLKLSSVTAADTAVYYCAR------------------

X92259|IGHV4-39*03| STYYNPSLKSRVTISVDTSKNQFSLKLSSVTAADTAVYY---------------------

AM940222|IGHV4-39*07| STYYNPSLKSRVTISVDTSKNQFSLKLSSVTAADTAVYYCAR------------------

X92250|IGHV4-61*04| STNYNPSLKSRVTISVDTSKNQFSLKLSSVTAD-TAVYY---------------------

AB019437|IGHV4-61*08| STNYNPSLKSRVTISVDTSKNQFSLKLSSVTAADTAVYYCAR------------------

M29811|IGHV4-61*01| STNYNPSLKSRVTISVDTSKNQFSLKLSSVTAADTAVYYCAR------------------

X92230|IGHV4-61*03| STNYNPSLKSRVTISVDTSKNHFSLKLSSVTAADTAVYYCAR------------------

KC713946|IGHV4-30-4*07| STYYNPSLKSRVTISVDTSKNQFSLKLSSVTAADTAVYYCAR------------------

X92229|IGHV4-30-2*03| STYYNPSLKSRVTISVDTSKNQFSLKLSSVTAADTAVYYCAR------------------

KC713944|IGHV4-30-2*06| STYYNPSLKSRVTISVDRSKNQFSLKLSSVTAADTAVYYCAR------------------

HM855593|IGHV4-30-2*05| STYYNPSLKSRVTISVDTSKNQFSLKLSSVTAADTAVYYCAR------------------

L10089|IGHV4-30-2*01| STYYNPSLKSRVTISVDRSKNQFSLKLSSVTAADTAVYYCAR------------------

M95122|IGHV4-30-2*02| STYYNPSLKSRVTISVDRSKNQFSLKLSSVTAADTAVYYCA-------------------

L10097|IGHV4-61*02| STNYNPSLKSRVTISVDTSKNQFSLKLSSVTAADTAVYYCAR------------------

Z14235|IGHV4-31*10| STYYNPSLKSRVTISVDPSKNQFSLKPSSVTAADTAVDYCAR------------------

M95121|IGHV4-31*05| STYYNPSLKSRVTISVDTSKNQFSLKLSSVTA-DAAVYYCA-------------------

X92273|IGHV4-31*09| STYYNPSLKSRVTISVDKSKNQFSLKLSSVTAADTAVYY---------------------

X92270|IGHV4-31*06| STYYNPSLKSRVTISVDTSKNQFSLKLSSVTAADTAVYY---------------------

M95120|IGHV4-31*04| STYYNPSLKSRVTISVDTSKNQFSLKLSSVTAADTAVYYCA-------------------

L10098|IGHV4-31*01| STYYNPSLKSLVTISVDTSKNQFSLKLSSVTAADTAVYYCAR------------------

M99683|IGHV4-31*02| STYYNPSLKSRVTISVDTSKNQFSLKLSSVTAADTAVYYCAR------------------

Z14237|IGHV4-31*03| STYYNPSLKSRVTISVDTSKNQFSLKLSSVTAADTAVYYCAR------------------

X92271|IGHV4-31*07| STYYNPSLKSRVTISVDTSKNQFSLKLSSVTAADTAVYY---------------------

X92272|IGHV4-31*08| STYYNPSLKSRVTISVDTSKNQFSLKLSSVTAADTAVYY---------------------

X92275|IGHV4-30-4*04| STYYNPSLKSRVTISVDTSKNQFSLKLSSVTAADTAVYY---------------------

Z14239|IGHV4-30-4*02| STYYNPSLKSRVTISVDTSKNQFSLKLSSVTAADTAVYYCAR------------------

Z14238|IGHV4-30-4*01| STYYNPSLKSRVTISVDTSKNQFSLKLSSVTAADTAVYYCAR------------------

X92274|IGHV4-30-4*03| STYYNPSLKSRVTISVDTSKNQFSLKLSSVTAADTAVYY---------------------

HM855782|IGHV4-28*06| STNYNPSLKSRVTMSVDTSKNQFSLKLSSVTALDTAVYYCAR------------------

X05714|IGHV4-28*01| STYYNPSLKSRVTMSVDTSKNQFSLKLSSVTAVDTAVYYCAR------------------

X92233|IGHV4-28*03| STYYNPSLKSRVTMSVDTSKNQFSLKLSSVTAVDTAVYYCAR------------------

KC713936|IGHV4-28*07| STYYNPSLKSRVTMSVDTSKNQFSLKLSSVTAVDTAVYYCAR------------------

X56358|IGHV4-28*04| STYYNPSLKSRVTMSVDTSKNQFSLKLSSVTAVDTGVYYCAR------------------

M83133|IGHV4-28*02| SIYYNPSLKSRVTMSVDTSKNQFSLKLSSVTAVDTAVYYCAR------------------

HM855339|IGHV4-28*05| SIYYNPSLKSRVTMSVDTSKNQFSLKLSSVTAVDTAVYYCAR------------------

Z12367|IGHV4-38-2*01| STYYNPSLKSRVTISVDTSKNQFSLKLSSVTAADTAVYYCAR------------------

AC233755|IGHV4-38-2*02| STYYNPSLKSRVTISVDTSKNQFSLKLSSVTAADTAVYYCAR------------------

Z14243|IGHV4-59*10| STNYNPSLKSRVTMSVDTSKNQFSLKLSSVTAADTAVYYCAR------------------

M95119|IGHV4-59*06| STYYNPSLKSRVTISVDTSKNQFSLKLSSVTAADTAVYYCA-------------------

X62112|IGHV4-4*07| STNYNPSLKSRVTMSVDTSKNQFSLKLSSVTAADTAVYYCAR------------------

M95118|IGHV4-59*05| STYYNPSLKSRVTISVDTSKNQFSLKLSSVTAADTAVYYCA-------------------

M95117|IGHV4-59*04| STYYNPSLKSRVTMSVDTSKNQFSLKLSSVTAADTAVYYCA-------------------

X56360|IGHV4-59*07| STNYNPSLKSRVTISVDTSKNQFSLKLSSVTAADTAVYYCAR------------------

M29812|IGHV4-59*02| STNYNPSLKSRVTISVDTSKNQFSLKLSSVTAADTAVYYCAR------------------

KC713942|IGHV4-4*08| STNYNPSLKSRVTISVDTSKNQFSLKLSSVTAADTAVYYCAR------------------

AB019438|IGHV4-59*01| STNYNPSLKSRVTISVDTSKNQFSLKLSSVTAADTAVYYCAR------------------

M95114|IGHV4-59*03| STNYNPSLKSRVTISVDTSKNQFSLKLSSVTAADTAVYYCA-------------------

HM855471|IGHV4-59*08| STNYNPSLKSRVTISVDTSKNQFSLKLSSVTAADTAVYYCAR------------------

. * ... :: * **.:. :. *: : :.:

**Supplementary Data 13**. Multiple sequence alignment of available commercial and IMGT heavy chain family for VH4 framework determination. Framework are chosen based on similarity among its family members highlighted in green.

**VH5 Framework Determination**

X92227|IGHV5-10-1*01| EVQLVQSGAEVKKPGESLRISCKGSGYSFTSYWISWVRQMPGKGLEWMGRIDPSDSYTNY

X92279|IGHV5-10-1*02| EVQLVQSGAEVKKPGESLRISCKGSGYSFTSYWISWVRQMPGKGLEWMGRIDPSDSYTNY

X56375|IGHV5-10-1*03| EVQLVQSGAEVKKPGESLRISCKGSGYSFTSYWISWVRQMPGKGLEWMGRIDPSDSYTNY

X56376|IGHV5-10-1*04| EVQLVQSGAEVKKPGESLRISCKGSGYSFTSYWISWVRQMPGKGLEWMGRIDPSDSYTNY

X56367|IGHV5-51*04| EVQLVQSGAEVKKPGESLKISCKGSGYSFTSYWIGWVRQMPGKGLEWMGIIYPGDSDTRY

M99686|IGHV5-51*01| EVQLVQSGAEVKKPGESLKISCKGSGYSFTSYWIGWVRQMPGKGLEWMGIIYPGDSDTRY

X56368|IGHV5-51*03| EVQLVQSGAEVKKPGESLKISCKGSGYSFTSYWIGWVRQMPGKGLEWMGIIYPGDSDTRY

M18806|IGHV5-51*02| EVQLVQSGAEVKKPGESLKISCKGSGYSFTSYWTGWVRQMPGKGLEWMGIIYPGDSDTRY

******************:************** .************** * *.** *.*

X92227|IGHV5-10-1*01| SPSFQGHVTISADKSISTAYLQWSSLKASDTAMYYCAR

X92279|IGHV5-10-1*02| SPSFQGHVTISADKSISTAYLQWSSLKASDTAMYYCAR

X56375|IGHV5-10-1*03| SPSFQGHVTISADKSISTAYLQWSSLKASDTAMYYCAR

X56376|IGHV5-10-1*04| SPSFQGQVTISADKSISTAYLQWSSLKASDTAMYYCAR

X56367|IGHV5-51*04| SPSFQGQVTISADKPISTAYLQWSSLKASDTAMYYCAR

M99686|IGHV5-51*01| SPSFQGQVTISADKSISTAYLQWSSLKASDTAMYYCAR

X56368|IGHV5-51*03| SPSFQGQVTISADKSISTAYLQWSSLKASDTAMYYCAR

M18806|IGHV5-51*02| SPSFQGQVTISADKSISTAYLQWSSLKASDTAMYYCAR

******:******* ***********************

**Supplementary Data 14**. Multiple sequence alignment of available IMGT heavy chain family for VH5 framework determination. Framework are chosen based on similarity among its family members highlighted in green.

**VH6 Framework Determination**

J04097|IGHV6-1*01| QVQLQQSGPGLVKPSQTLSLTCAISGDSVSSNSAAWNWIRQSPSRGLEWLGRTYYRSKWY

Z14223|IGHV6-1*02| QVQLQQSGPGLVKPSQTLSLTCAISGDSVSSNSAAWNWIRQSPSRGLEWLGRTYYRSKWY

************************************************************

J04097|IGHV6-1*01| NDYAVSVKSRITINPDTSKNQFSLQLNSVTPEDTAVYYCAR

Z14223|IGHV6-1*02| NDYAVSVKSRITINPDTSKNQFSLQLNSVTPEDTAVYYCAR

*****************************************

**Supplementary Data 15**. Multiple sequence alignment of available IMGT heavy chain family for VH5 framework determination. Framework are chosen based on similarity among its family members highlighted in green.

**VH7 Framework Determination**

X92290|IGHV7-4-1*03| QVQLVQSGSELKKPGASVKVSCKASGYTFTSYAMNWVRQAPGQGLEWMGWINTNTGNPTY

HM855361|IGHV7-4-1*05| QVQLVQSGSELKKPGASVKVSCKASGYTFTSYAMNWVRQAPGQGLEWMGWINTNTGNPTY

HM855485|IGHV7-4-1*04| QVQLVQSGSELKKPGASVKVSCKASGYTFTSYAMNWVRQAPGQGLEWMGWINTNTGNPTY

L10057|IGHV7-4-1*01| QVQLVQSGSELKKPGASVKVSCKASGYTFTSYAMNWVRQAPGQGLEWMGWINTNTGNPTY

X62110|IGHV7-4-1*02| QVQLVQSGSELKKPGASVKVSCKASGYTFTSYAMNWVRQAPGQGLEWMGWINTNTGNPTY

Ranibizumab EVQLVESGGGLVQPGGSLRLSCAASGYDFTHYGMNWVRQAPGKGLEWVGWINTYTGEPTY

Bevacizumab EVQLVESGGGLVQPGGSLRLSCAASGYTFTNYGMNWVRQAPGKGLEWVGWINTYTGEPTY

Oportuzumab ------------------------------------VKQAPGKGLEWMGWINTYTGESTY

*:****:****:***** **: **

X92290|IGHV7-4-1*03| AQGFTGRFVFSLDTSVSTAYLQISTLKAEDT-----------------------------

HM855361|IGHV7-4-1*05| AQGFTGRFVFSLDTSVSMAYLQISSLKAEDTAVCYCAR----------------------

HM855485|IGHV7-4-1*04| AQGFTGRFVFSLDTSVSMAYLQISSLKAEDTAVYYCAR----------------------

L10057|IGHV7-4-1*01| AQGFTGRFVFSLDTSVSTAYLQICSLKAEDTAVYYCAR----------------------

X62110|IGHV7-4-1*02| AQGFTGRFVFSLDTSVSTAYLQISSLKAEDTAVYYCAR----------------------

Ranibizumab AADFKRRFTFSLDTSKSTAYLQMNSLRAEDTAVYYCAKYPYYYGTSHWYFDVWGQGTLVT

Bevacizumab AADFKRRFTFSLDTSKSTAYLQMNSLRAEDTAVYYCAKYPHYYGSSHWYFDVWGQGTLVT

Oportuzumab ADSFKGRFTFSLDTSASAAYLQINSLRAEDTAVYYCARFAIK-------GDYWGQGTLLT

* *. **.****** * ****: :*:****

**Supplementary Data 16**. Multiple sequence alignment of available commercial and IMGT heavy chain family for VH7 framework determination. Framework are chosen based on similarity among its family members highlighted in green.
